# Reciprocal Genome Projection Reveals Reference-Conditioned Functional Variation in Clinical *Aspergillus* Section *Nigri* Isolates

**DOI:** 10.3390/pathogens15090941

**Published:** 2026-09-04

**Authors:** Siphiwengesihle Kuhle Silindile Mbamali, Nikwando Mohlomi, Magriet van der Nest, Nokuthula Peace Mchunu, Kugenthiren Permaul

**Affiliations:** 1 Department of Biotechnology and Food Science, Faculty of Applied Sciences, Durban University of Technology, Durban 4001, South Africa; 22290625@dut4life.ac.za (N.M.); kugen@dut.ac.za (K.P.); 2 Forestry and Agricultural Biotechnology Institute (FABI), Department of Biochemistry, Genetics and Microbiology, Faculty of Natural and Agricultural Sciences, University of Pretoria, Pretoria 0028, South Africa; 3 School of Life Science, Westville, College of Agriculture, Engineering and Science, University of KwaZulu-Natal, Durban 4000, South Africa; np.mchunu@nrf.ac.za; 4 National Research Foundation, Meiring Naude Road, Brummeria, Pretoria 0001, South Africa

**Keywords:** comparative genomics, reference genome, *Aspergillus niger*, *Aspergillus welwitschiae*, *Aspergillus* section-*Nigri*

## Abstract

Closely related *Aspergillus* section *Nigri* species are difficult to resolve taxonomically, while short-read comparisons may be influenced by reference-genome choice. We applied reciprocal genome projection to 31 whole-genome sequencing libraries representing South African clinical section-*Nigri* isolates, Japanese and USA *A. niger*-labeled isolates, and *A. welwitschiae*-labeled isolates. Reads were aligned independently to *A. niger* ATCC 1015 and *A. welwitschiae* Aspwel1, followed by reference-specific differential-representation and KEGG analyses. Species-level classification showed substantial overlap between *A. niger* and *A. welwitschiae*. The *A. welwitschiae*–South African comparison identified 44 and 59 effect-qualified features under the respective projections, including 20 reciprocal feature pairs defining a reference-robust *A. welwitschiae*-associated core. The clinical Japanese–South African comparison identified 116 and 127 features, with 14 concordant KEGG orthologs involving sterol modification, protein prenylation, cofactor metabolism, redox processing, cell-wall turnover and ribosome maturation. No pathway remained significant after false discovery-rate correction; nominal pathway signals were reference-sensitive. Reciprocal projection, therefore, recovered localized cohort-associated genomic differentiation while identifying reference-conditioned effects. These findings represent differential genomic-read representation and do not independently establish copy-number variation, pathway activity, virulence or antifungal resistance.

## 1. Introduction

Aspergillus section *Nigri* comprises genetically diverse black *aspergilli* with substantial industrial, food-safety and clinical relevance [1]. The section has historically included more than two dozen described species, including *A. niger*, *A. tubingensis*, *A. luchuensis*, *A. carbonarius* and *A. brasiliensis*. Broad biseriate and uniseriate groups have been recognized within the section and differ in sexual states, sclerotium formation and secondary metabolite production [1]. Several species are exploited for organic acid and extracellular enzyme production [2], whereas others contribute to food spoilage and mycotoxin contamination [1]. Clinically relevant and cryptic section *Nigri* species have also been recovered from human infections, where accurate species-level identification can be difficult [3].

This taxonomic complexity is particularly relevant to *A. niger* and *A. welwitschiae*. Recent species delimitation within series *Nigri* proposed a reduced set of accepted species and treated *A. welwitschiae* as a synonym of *A. niger* [4]. Public repositories, nevertheless, continue to contain genomes labeled under both names. A phylogenomic analysis of 710 publicly available *Aspergillus* genomes estimated that nearly 8% were misidentified, with the *A. niger* and *A. welwitschiae* relationship representing a notable source of ambiguity [5]. Considerable structure also exists within *A. niger* sensu stricto, where analysis of 24 strains identified three genomic clades and an average of 6.1 ± 2.0 variants per kilobase [6]. Comparative genomics has further revealed extensive variation in gene content and secondary metabolite capacity across section *Nigri* [1]. Consequently, isolates carrying the same species label may differ appreciably in sequence similarity, gene complement and functional genomic representation.

Most short-read comparative workflows align samples to a single linear reference genome. In comparisons involving divergent lineages, reference selection is not analytically neutral. Reads from loci that differ from the selected reference may align less efficiently, whereas more similar loci may appear disproportionately represented. Gene-associated WGS counts can, therefore, reflect biological variation in gene presence, dosage and sequence divergence, together with technical factors such as local mappability and annotation correspondence. This form of reference bias is recognized in short-read alignment but is difficult to separate from cohort-associated variation in a conventional single reference analysis [7]. We, therefore, applied reciprocal genome projection to 31 paired-end WGS libraries representing South African clinical section *Nigri* isolates, Japan-labeled and USA-labeled *A. niger* isolates and *A. welwitschiae*-labeled isolates. Each library was independently projected onto the *A. niger* ATCC 1015 and *A. welwitschiae* reference assemblies, with the resulting count matrices analyzed separately and compared through reciprocal sequence correspondence and pathway annotation.

The primary objective was to determine whether reciprocal genome projection could differentiate *A. welwitschiae*-labeled isolates from the *A. niger* cohorts despite substantial overlap in read-level taxonomic assignments. Within this broader species boundary analysis, the clinical JPY versus ZAR comparison examined cohort- or lineage-associated differentiation among clinical section *Nigri* isolates, while the USA cohort provided additional *A. niger* genomic context. Observed differences were interpreted as differential genomic representation rather than differential expression, formal copy-number variation or definitive species reassignment. By separating signals consistent across both references from effects conditioned by reference choice, this study provides a conservative framework for resolving taxonomic and functional genomic variation, including differences affecting terpenoid biosynthesis and other adaptive pathways, among closely related black aspergilli.

## 2. Materials and Methods

### 2.1. Study Design and Analytical Interpretation

South African clinical black *Aspergillus* isolates were recovered from human clinical samples obtained through Inkosi Albert Luthuli Central Hospital, Durban, KwaZulu-Natal, South Africa. Patient identifiers were omitted before analysis to maintain ethical confidentiality. Isolates were cultured on malt extract agar (MEA) for 3 days at 37 °C [8]. Genomic DNA was extracted from 8 clinical isolates (PRJNA1475450) using the E.Z.N.A. Plant & Fungal DNA Kit (Omega Bio-tek, Norcross, GA, USA), according to the manufacturer’s protocol. DNA quantity and purity were assessed using a NanoPhotometer N60 spectrophotometer (Implen, Munich, Germany). High-quality genomic DNA was submitted to the Biotechnology Platform of the Agricultural Research Council, Onderstepoort, South Africa, for whole-genome sequencing. DNA was sheared to an average fragment length of approximately 270 bp, adapter-ligated using KAPA or Nextera library-preparation chemistry, size-selected and sequenced on an Illumina MiSeq platform using 2 × 300 bp paired-end reads. The sequencing generated paired-end FASTQ files for downstream taxonomic classification, reference-guided mapping, variant calling and comparative genomic analyses.

Paired-end whole-genome sequencing libraries were assigned to four operational cohorts: South African clinical section-*Nigri* isolates (ZAR_Nigri; *n* = 8), publicly available Japan-labeled *A. niger* isolates (JPY; *n* = 8), USA-labeled *A. niger* isolates (USA; *n* = 8), and *A. welwitschiae*-labeled isolates (Awel; *n* = 7). Sample accessions are provided in Table 1. Each library represented one biological isolate; sample identifiers were reconciled with the BAM-derived manifest before cohort assignment. Pairwise differential-abundance analyses were conducted independently for each reference-genome projection. ZAR_Nigri was specified as the reference condition, while the comparator cohort was specified as the primary contrast condition. Thus, for the JPY/ZAR_Nigri comparison, positive log_2_ fold changes represent higher gene-associated genomic abundance in JPY and negative values represent higher abundance in ZAR_Nigri. Likewise, for the Awel ZAR_Nigri comparison, positive values represent higher abundance in Awel; negative values represent higher abundance in ZAR_Nigri.

### 2.2. Read Preprocessing, Taxonomic Screening and Alignment

Raw paired-end Illumina reads were assessed for sequencing quality before and after adapter and quality trimming using FastQC (v0.12.1) and MultiQC (v1.35) [11,12,13]. Trimming was performed with Trimmomatic (v0.39) through the KBase (v5.5.1) platform to remove Illumina adapter sequences, low-quality terminal bases and short reads [14,15]. Only read pairs for which both mates passed quality filtering were retained for downstream analysis. The cleaned reads were screened with Kaiju against the NCBI RefSeq protein database to identify major contaminants and to support the operational assignment of samples to the study cohorts [16]. This screening was used as a quality-control and classification step rather than as a definitive species-identification procedure. For the primary analysis, reads were aligned to the *Aspergillus niger* ATCC 1015 ASPNI v3.0 reference genome and its corresponding gene annotation (GenBank GCA_000230395.2) [17]. Alignment was performed with BWA-MEM (v2.3), after which the resulting files were sorted and indexed with SAMtools (v1.24) [18,19]. Alignment quality and genome coverage were evaluated with Qualimap (v2.3) and MultiQC (v1.35) [13,20]. To assess the sensitivity of the results to the reference-genome choice, the same cleaned reads were also aligned independently to the *A. welwitschiae* Aspwel1 reference genome (RefSeq GCF_003344945.1). Count matrices generated from the ASPNI and Aspwel1 projections were analyzed separately and were not combined. Because the principal cross-group differential-abundance table contains ASPNIDRAFT gene identifiers, that table represents the ASPNI-centered analysis.

### 2.3. Annotation-Defined WGS Gene Counting

BAM files were checked for integrity, coordinate sorting, indexing and consistency with the corresponding reference genome and GFF3 annotation. Gene coordinates were extracted from the annotation and converted into genomic intervals using Bioconductor tools [21,22]. The ASPNI annotation initially contained 10,947 gene intervals.

### 2.4. Differential Genomic Representation

Gene-associated count matrices generated under the *A. niger*-centered and *A. welwitschiae*-centered projections were analyzed independently in a simple one-factor cohort design [23,24]. Library-size differences were normalized using the relative log expression method (RLE), and robust dispersion estimation was enabled. Cohort effects were tested using a negative-binomial generalized linear model likelihood-ratio test. For the clinical JPY vs. ZAR_Nigri analysis, JPY was entered as the primary contrast condition and ZAR as the reference condition in both genome projections. The *A. niger*-centered matrix contained 10,947 initial features, of which 262 were filtered, leaving 10,685 features; the reciprocal projection contained 13,950 initial features, of which 418 were filtered, leaving 13,532 features. For the Awel vs. ZAR_Nigri analysis, Awel was entered as the primary contrast condition and ZAR_Nigri as the reference condition. The 13,950-feature projection retained 13,430 features after automatic filtering, whereas the 10,947-feature projection retained 10,617 features. Both analyses used RLE normalization, a simple cohort design, robust estimation and a GLM likelihood-ratio test. Multiple testing was controlled using the Benjamini–Hochberg false-discovery rate (FDR) [25]. Loci with FDR < 0.05 were regarded as statistically supported.

### 2.5. Functional Annotation and Pathway Enrichment

ASPNI features were functionally annotated with eggNOG-mapper with gene/contig input, an Ascomycota taxonomic scope (NCBI taxon 4890), all target orthologs and a minimum accepted hit E value of 1 × 10^−3^ [26,27]. KEGG Orthology identifiers were linked through eggNOG-mapper; Enzyme Commission annotations supplied additional direct links to generic KEGG pathways [28]. KEGG was enabled, whereas Reactome and Gramene were disabled. The included KEGG categories were: Metabolism, Genetic Information Processing, Environmental Information Processing, Cellular Processes, Organismal Systems and Drug Development. Human Diseases was excluded. Combined pathway analysis evaluated threshold-based over-representation and whole-list rank enrichment [29]. For the two-tailed Fisher analysis, the reference universe comprised all input features linked to at least one KEGG pathway, and the test set comprised loci carrying either directional tag. Pathway-level Fisher results were filtered at FDR < 0.05.

Gene-set enrichment analysis was performed in pre-ranked mode (pre-ranked GSEA) using OmicsBox (v4.3.1) [29,30], following the method of Subramanian et al. [31]. All modeled features were ranked by sign(log_2_FC) × −log_10_(*p* value), ordering genes from those most strongly associated with the comparator cohort (positive ranks) to those most associated with the reference cohort (negative ranks) [31]. This ranking combines effect direction and statistical evidence into a single continuous score. Enrichment was assessed using the Classic statistic with 1000 gene-set permutations, restricted to gene sets containing 15–500 annotated features. Given the limited annotation depth and the absence of FDR-corrected pathway-level support from the Fisher analysis, GSEA results were treated as hypothesis-generating and filtered at a lenient q ≤ 0.25 threshold. TOP and BOTTOM designations indicate enrichment at the positive and negative ends of the ranked list, respectively [29,30].

### 2.6. Targeted Evidence Assessment of Functional Assignments

To assess the evidential support for the functional assignments highlighted in the clinical JPY versus ZAR_Nigri comparison, the nine reference concordant KEGG ortholog assignments explicitly interpreted in the Results were subjected to targeted evidence assessment. Exact ASPNI and Aspwel1 locus identifiers were cross-checked against the corresponding eggNOG mapper outputs; annotation E values were recorded where exact locus matches were recovered. KEGG Orthology records were inspected for functional definitions, Enzyme Commission assignments and the associated primary literature. Primary studies were then examined for genetic, biochemical, structural, mutational or heterologous expression evidence supporting the assigned molecular function. Experimental evidence from the characterized orthologues was used to assess support for the functional annotation only and was not interpreted as evidence of altered transcription, enzyme activity, pathway flux, virulence or antifungal susceptibility in the isolates analyzed here (See Supporting data File S1).

## 3. Results

### 3.1. Mixed Species-Level Assignments Supported Reciprocal Genome Projection

**Figure 1:**
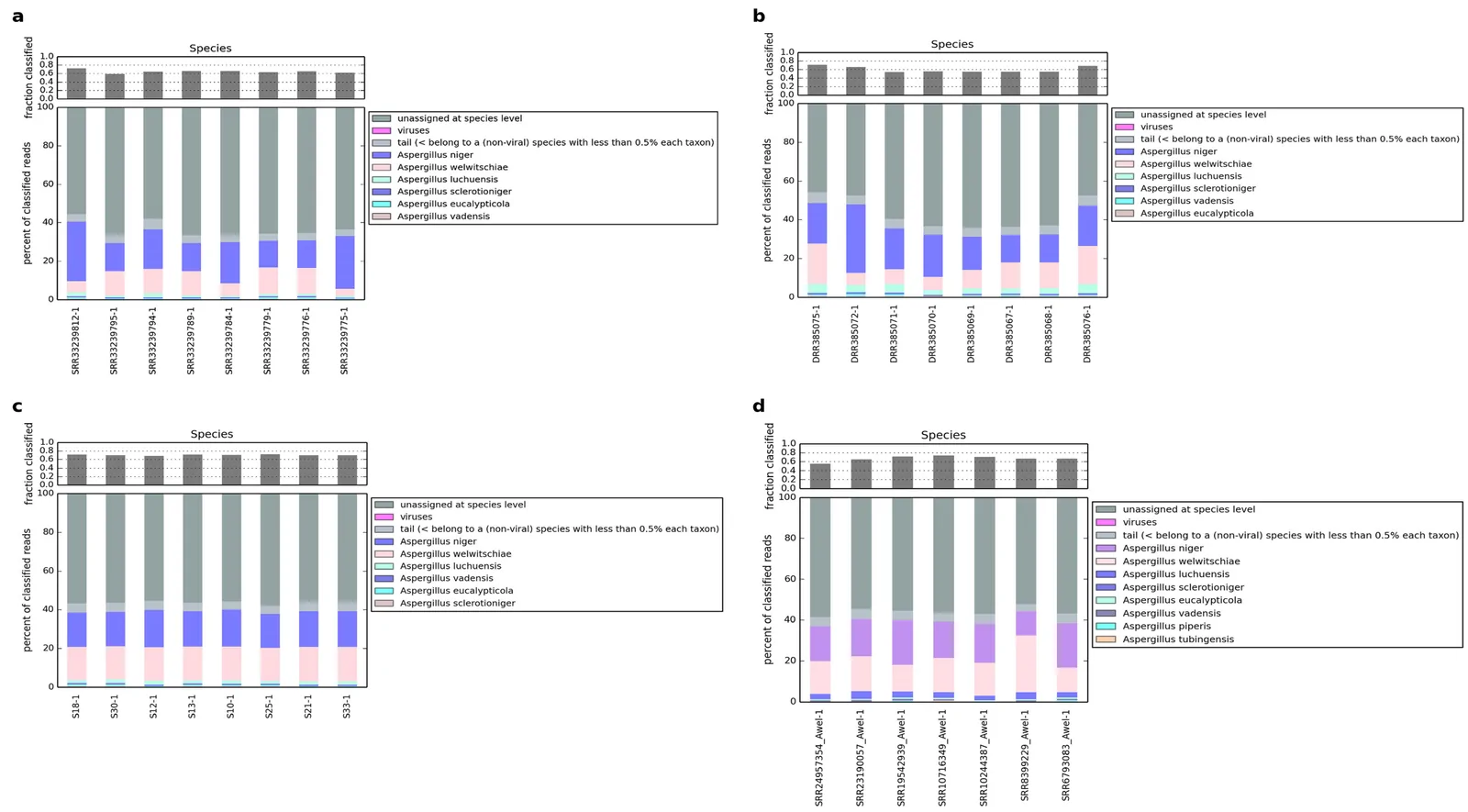
Species-level taxonomic composition of sequencing reads across four *Aspergillus* section Nigri cohorts. Kaiju-derived profiles are shown for: (**a**) the USA cohort comprising NCBI-deposited *A. niger* datasets; (**b**) the Japanese cohort comprising NCBI-deposited *A. niger* datasets; (**c**) the South African clinical cohort; and (**d**) NCBI-deposited *A. welwitschiae* datasets.

The fraction of reads classified varied moderately among cohorts. It was approximately 0.64–0.68 in the local ZAR_Nigri isolates, 0.56–0.70 in the USA cohort, 0.48–0.66 in the JPY cohort and 0.53–0.71 in the Awel cohort. The JPY libraries, therefore, showed the broadest variation and generally the lowest classified fractions, whereas the local isolates produced comparatively uniform values. Reads unassigned at the species level formed the largest component of the classified-read profiles, representing approximately 54–57% in ZAR_Nigri, 55–66% in USA, 44–63% in JPY and 52–58% in Awel. Among named species assignments, *A. niger* and *A. welwitschiae* dominated every cohort. The local isolates showed particularly consistent and nearly balanced profiles, with approximately 16–18% of classified reads assigned to *A. niger* and 16–17% to *A. welwitschiae*. In the USA cohort, *A. niger* assignments varied from approximately 13% to 30%, compared with about 2–13% for *A. welwitschiae*. SRR33239812 and SRR33239775 showed the strongest relative *A. niger* components. The JPY cohort was more heterogeneous, with approximately 13–34% assigned to *A. niger* and 5–20% to *A. welwitschiae*. DRR385072 was comparatively *A. niger*-enriched, whereas DRR385075 and DRR385076 contained nearly balanced contributions from the two taxa. The Awel libraries contained approximately 11–22% *A. niger*-assigned reads and 11–28% *A. welwitschiae*-assigned reads (Figure 1). SRR8399229 showed the strongest *A. welwitschiae* signal, at approximately 28% compared with approximately 11% for *A. niger*. Conversely, SRR19542939 and SRR6793083 contained larger *A. niger* than *A. welwitschiae* components. Other named section-Nigri taxa and the aggregated low-abundance tail accounted for the remaining classified reads.

Thus, species-level read assignments did not partition cleanly according to the public cohort labels. These protein-level classifications were used to support section-Nigri membership and detect broad taxonomic patterns, not to establish definitive isolate identities. The mixed assignments provided the rationale for projecting all libraries independently onto ASPNI and Aspwel1 and for conducting differential genomic-representation and pathway analyses separately within each reference coordinate system.

### 3.2. Read Alignment and BAM Quality Supported Downstream Counting

The study dataset comprised 31 paired-end libraries assigned to ZAR_Nigri (*n* = 8), JPY (*n* = 8), USA (*n* = 8) and Awel (*n* = 7). Coordinate-sorted BAMs used for counting passed integrity, indexing and reference-dictionary checks. In the complete Aspwel1 alignment, median mapping rates were 92.61% for ZAR_Nigri, 88.29% for JPY, 92.23% for USA and 90.46% for Awel. Median mean depths were 26.80×, 53.36×, 128.07× and 102.75×, respectively. In the ASPNI report, the complete ZAR_Nigri, USA and Awel cohorts had median mapping rates of 90.32%, 91.72% and 86.66%, with corresponding median depths of 27.88×, 139.54× and 106.99×. Mean mapping-quality scores remained high, with cohort medians of approximately 58.9–59.0 under ASPNI (Table A1, see Appendix A) and 50.9–53.2 under Aspwel1 (Table A2). Across the available reciprocal alignments, individual libraries retained at least 85% mapped reads and approximately 14× mean genomic depth. Both-read recovery ranged from 83.9% to 95.2%, singleton fractions did not exceed 2.3%, supplementary alignments remained below 1.6% and mapped-base GC content ranged from 49.2% to 51.8%. No library, therefore, showed evidence of global alignment failure. Library depth and estimated duplicate representation were more heterogeneous, with duplicate fractions ranging from approximately 7.5% to 70.3% and generally higher values in the deeper USA and Awel libraries. Consequently, raw gene-span counts were not compared directly; reference-specific count matrices were normalized and analyzed using replicate-based robust dispersion modeling. Having established adequate alignment and BAM integrity, annotation-defined genomic feature counts were evaluated independently for each reference projection.

### 3.3. Differential Genomic Projections of Aspergillus welwitschiae and Aspergillus niger

**Figure 2:**
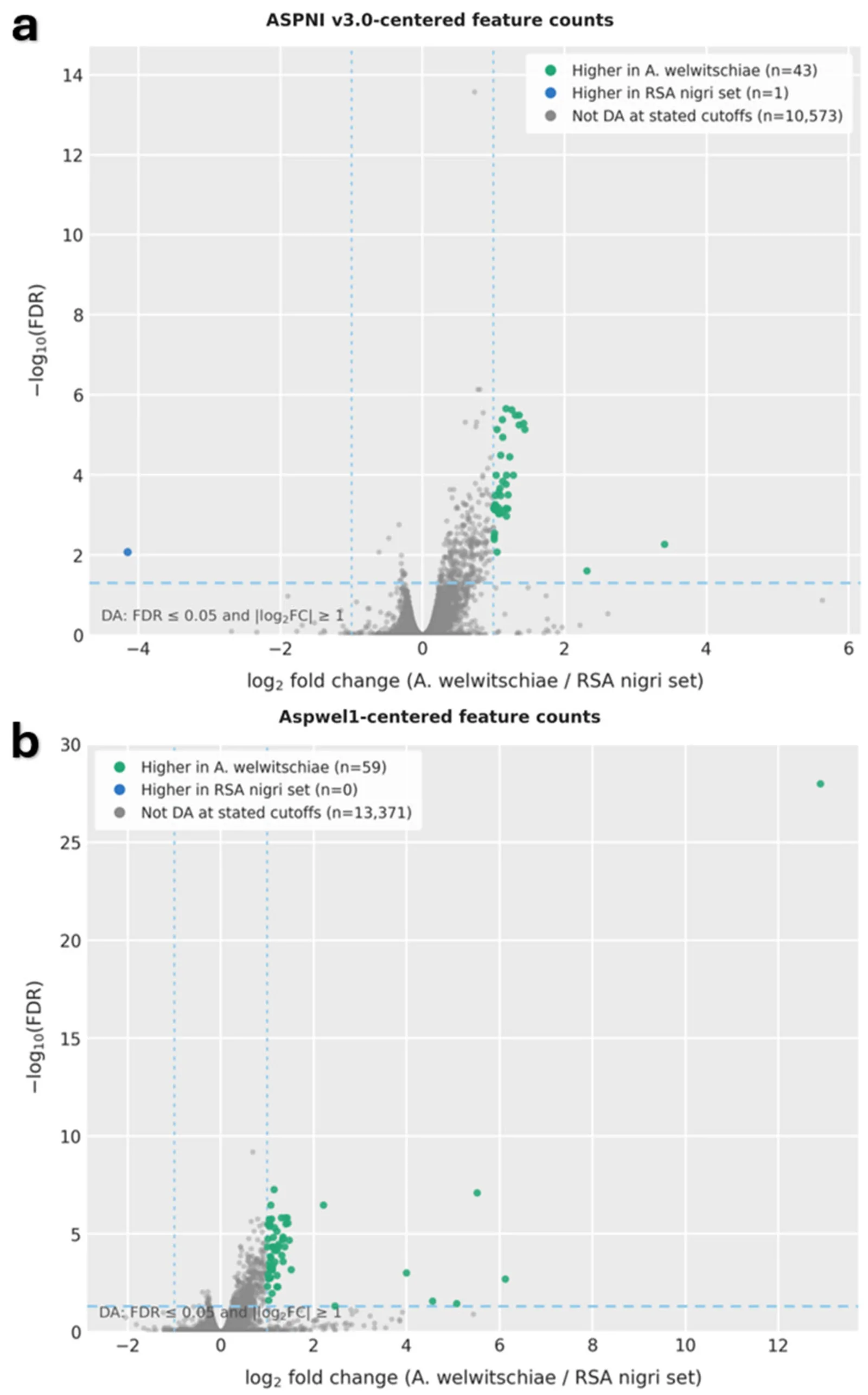
Differential gene-associated representation between the Awel and RSA section Nigri cohorts under two reference-genome projections. Volcano plots show edgeR results obtained from WGS-derived feature counts aligned to: (**a**) the ASPNI v3.0 reference; and (**b**) the Aspwel1 reference. Positive log_2_ fold changes indicate higher representation in JPY, whereas negative values indicate higher representation in RSA.

Unsupervised, independent projection of the same libraries onto the two reference genomes produced a strongly asymmetric, Awel-biased differential-representation signal. After automatic abundance filtering, 10,617 ASPNI-defined and 13,430 Aspwel1-defined gene features were tested. At FDR ≤ 0.05 alone, 350 ASPNI-centric features (3.30%) and 437 Aspwel1-centric features (3.25%) were significant. Most of these statistically resolved differences were nevertheless small: 306 of 350 ASPNI-centric features (87.4%) and 378 of 437 Aspwel1-centric features (86.5%) had |log_2_FC| < 1 and remained grey in the volcano plots (Figure 2). Application of the prespecified combined criterion of FDR ≤ 0.05 and |log_2_FC| ≥ 1, therefore, retained 44 ASPNI-centric features (0.41% of those tested) and 59 Aspwel1-centric features (0.44%). Directional asymmetry was already evident among all FDR-significant features, of which 335/350 (95.7%) and 422/437 (96.6%) had positive log_2_FC in the ASPNI- and Aspwel1-centric analyses, respectively. In contrast, the genome-wide median log_2_FC remained close to zero in both feature spaces (−0.013 and −0.015), arguing against a uniform shift across all loci. Within the ASPNI-centric feature space, 43 of the 44 effect-qualified features showed greater representation in Awel, whereas only `ASPNIDRAFT_51714` showed greater representation in the RSA nigri set (log_2_FC = −4.149; FDR = 0.00843), corresponding to an approximately 17.7-fold difference in the opposite direction. The positive ASPNI-centric effects were generally moderate: the median positive log_2_FC was 1.105, and 41 of the 44 total effect-qualified features had |log_2_FC| < 1.5. Two positive outliers stood above this compact band, `ASPNIDRAFT_45813` (log_2_FC = 3.407; FDR = 0.00546; approximately 10.6-fold) and `ASPNIDRAFT_123557` (log_2_FC = 2.316; FDR = 0.0247; approximately 5.0-fold). Thus, the ASPNI-centric result was characterized by a broad set of statistically supported twofold differences, two stronger Awel-associated features and a single high-effect feature associated with the RSA nigri set. The Aspwel1-centric result was entirely Awel-directed at the combined threshold: all 59 effect-qualified features had positive log_2_FC. Although the median effect remained moderate (log_2_FC = 1.153), this analysis contained a substantially heavier positive tail than the ASPNI-centric analysis, with eight features at log_2_FC ≥ 2, six at log_2_FC ≥ 3 and five at log_2_FC ≥ 4. The most extreme feature, `BDQ94DRAFT_164183`, encoded an annotated BTB/POZ domain-containing protein and had log_2_FC = 12.899 (FDR = 1.03 × 10^−28^). Importantly, this feature was not an isolated genomic point: it occurred with `BDQ94DRAFT_127417` (log_2_FC = 5.519; FDR = 8.05 × 10^−8^), `BDQ94DRAFT_164185` (log_2_FC = 4.558; FDR = 0.0270) and `BDQ94DRAFT_164188` (log_2_FC = 6.125; FDR = 0.00206) within a 9.8-kb interval on scaffold NW_020798779.1. The latter three genes are currently annotated as uncharacterized proteins. Their physical concentration suggests that the four gene-level signals may reflect a single regional event, such as differential presence, copy representation, or reference-dependent mappability, rather than four independent functional changes. Additional high-effect Aspwel1 loci occurred on separate scaffolds, including `BDQ94DRAFT_164253` (log_2_FC = 5.077; FDR = 0.0357), `BDQ94DRAFT_183128` (log_2_FC = 3.995; FDR = 9.79 × 10^−4^) and `BDQ94DRAFT_175094` (log_2_FC = 2.460; FDR = 0.0489). Despite the use of different reference-defined identifiers, a reciprocal 31-mer sequence–correspondence check linked 20 effect-qualified ASPNI/Aspwel1 feature pairs (canonical 31-mer Dice coefficients, 0.34–0.71). These paired loci define a reference-robust core of the Awel-associated signal, whereas the unmatched and regionally clustered features capture reference-conditioned differences. Functional interpretation remained limited by annotation depth. Forty-one of the 59 Aspwel1-centric effect-qualified features (69.5%) were annotated only as uncharacterized proteins. The annotated minority represented heterogeneous processes, including spliceosome-associated proteins (CWC21 and Saf4/Yju2 families), ribosome biogenesis (IPI1/TEX10), DNA repair (Dds20/Mei5), protein prenylation processing, alcohol dehydrogenase activity, DJ-1/PfpI-family function, endoplasmic-reticulum membrane-complex function (EMC6), chromosome segregation (DAD3 and APC13 families) and a Shr3-like secretory component. This dispersion did not support a single dominant functional program. Consistent with that locus-level pattern, no KEGG pathway passed Fisher FDR correction at 0.05 in either reference-conditioned Awel–versus–RSA comparison; directional GSEA TOP/BOTTOM labels were, therefore, not treated as evidence of pathway-level statistical significance.

### 3.4. Pathway-Level Variation Was Nominal

**Figure 3:**
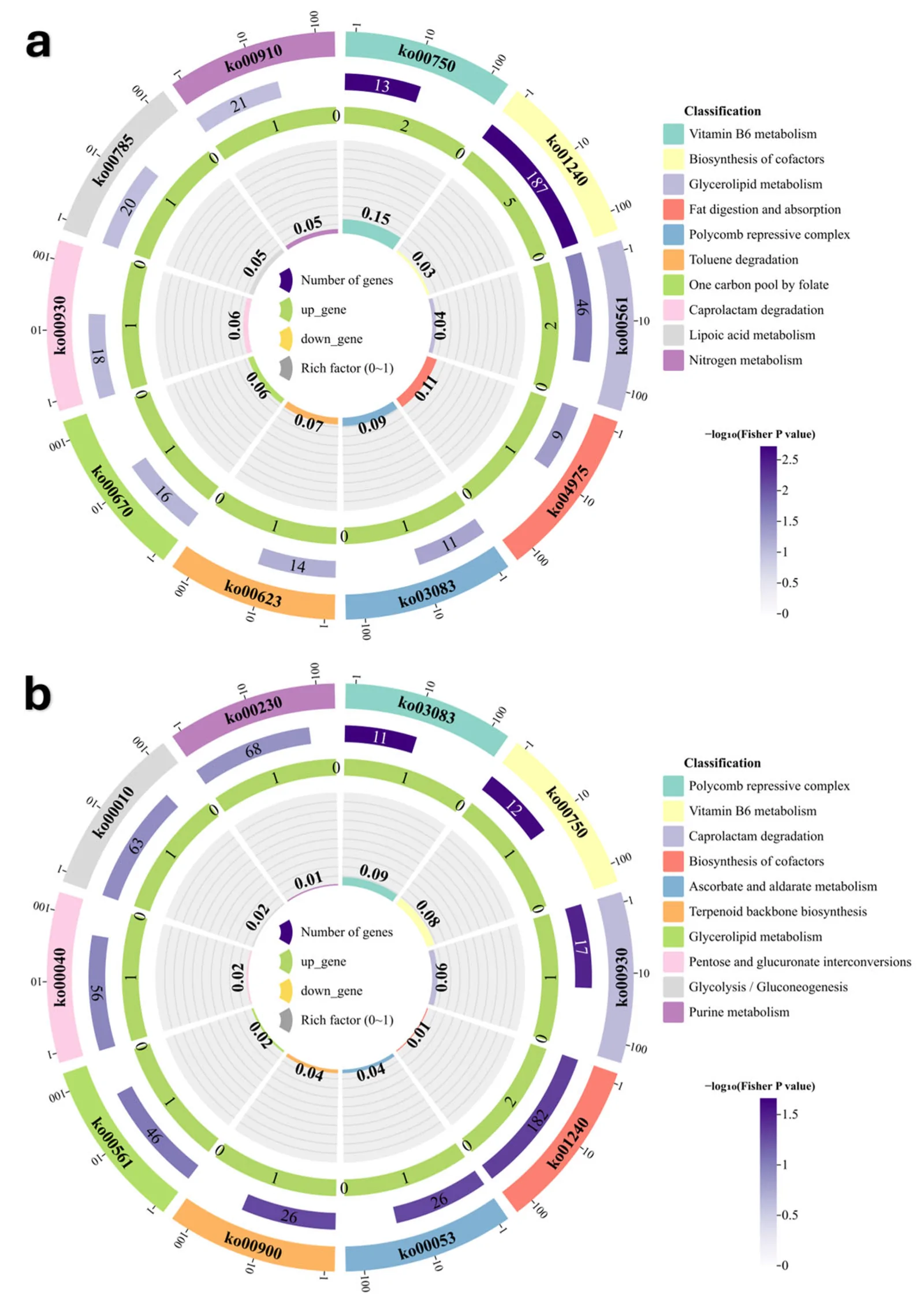
Fisher’s exact-test pathway enrichment for the Awel versus ZAR_Nigri comparison under the two reference-centered analytical approaches: (**a**) *Aspergillus welwitschiae*-centered analysis using the Aspwel1 reference genome; and (**b**) *Aspergillus niger*-centered analysis using the ASPNI v3.0 reference genome.

Two-tailed Fisher analysis evaluated 323 KEGG pathways in the ASPNI-centered projection and 322 in the Aspwel1-centered projection. Four pathways had nominal Fisher *p* < 0.05 under each reference, but none remained supported after multiple-testing correction (Figure 2). Under ASPNI (Figure 3a), the smallest nominal *p* values were observed for vitamin B6 metabolism (2 of 13 pathway-linked loci carrying positive tags, *p* = 0.00190), biosynthesis of cofactors (5/187, *p* = 0.00191), glycerolipid metabolism (2/46, *p* = 0.0229) and fat digestion and absorption (1/9, *p* = 0.0459). The minimum pathway-level FDR was 0.308. Under Aspwel1, nominal signals involved the Polycomb repressive complex (1/11, *p* = 0.0217), vitamin B6 metabolism (1/12, *p* = 0.0237), caprolactam degradation (1/17, *p* = 0.0334) and biosynthesis of cofactors (2/182, *p* = 0.0464). All Aspwel1 Fisher FDR values were 1.0. Every tagged locus contributing to these nominal pathway results was higher in Awel; no negatively tagged locus contributed to any of the pathways (Figure 3b). Vitamin B6 metabolism and biosynthesis of cofactors were, therefore, the only nominal Fisher signals reproduced under both reference projections. Vitamin B6 metabolism, biosynthesis of cofactors, glycerolipid metabolism, the Polycomb repressive complex and caprolactam degradation appeared among the ten lowest nominal *p* values under both references, although their ordering and nominal support varied. These results indicate limited recurrence of pathway labels but do not constitute FDR-supported pathway enrichment. Moreover, generic KEGG labels, such as “fat digestion and absorption”, identify shared ortholog sets and should not be interpreted as evidence of the corresponding organism-level physiology in *Aspergillus*.

Exploratory pre-ranked analysis provided broader directional context. Among gene sets eligible for GSEA, concordant positive ranking toward the Awel end was observed for glyoxylate and dicarboxylate metabolism (ASPNI/Aspwel1 NES = 2.45/2.83), biosynthesis of cofactors (2.26/2.84), peroxisome (2.37/2.08) and valine, leucine and isoleucine biosynthesis (2.40/2.15). Concordant negative ranking toward ZAR_Nigri included aminoacyl-tRNA biosynthesis (−2.31/−1.97), yeast cell cycle (−2.24/−2.06) and DNA replication (−2.00/−1.82). These NES patterns provide exploratory whole-list context and were not treated as confirmatory pathway evidence.

### 3.5. Differential Genomic Projections of Clinical Cohorts

**Figure 4:**
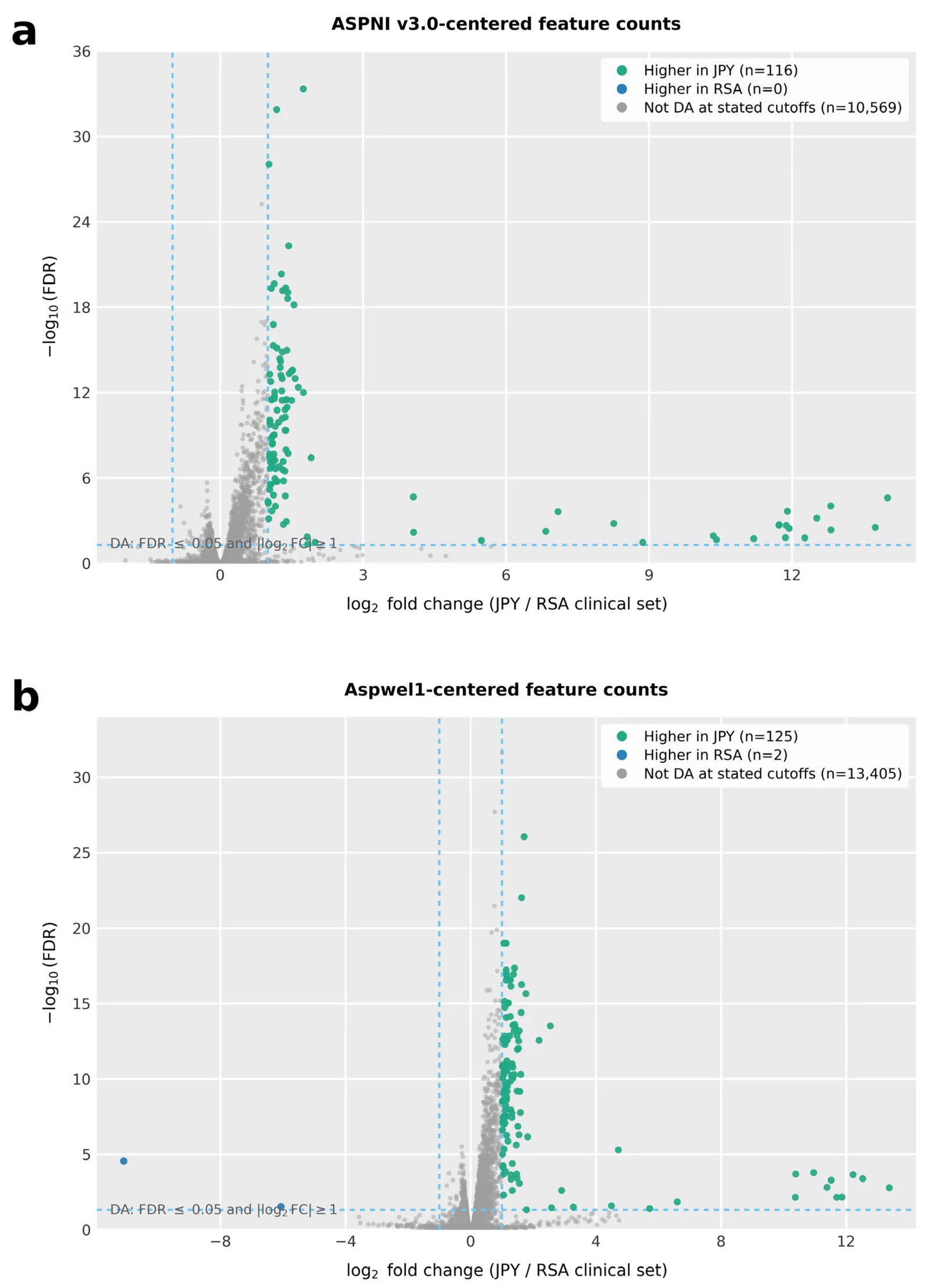
Differential gene-associated representation between the JPY and RSA clinical cohorts under two reference-genome projections. Volcano plots show edgeR results obtained from WGS-derived feature counts aligned to: (**a**) the ASPNI v3.0 reference; and (**b**) the Aspwel1 reference. Positive log_2_ fold changes indicate higher representation in JPY, whereas negative values indicate higher representation in RSA.

**Figure 5:**
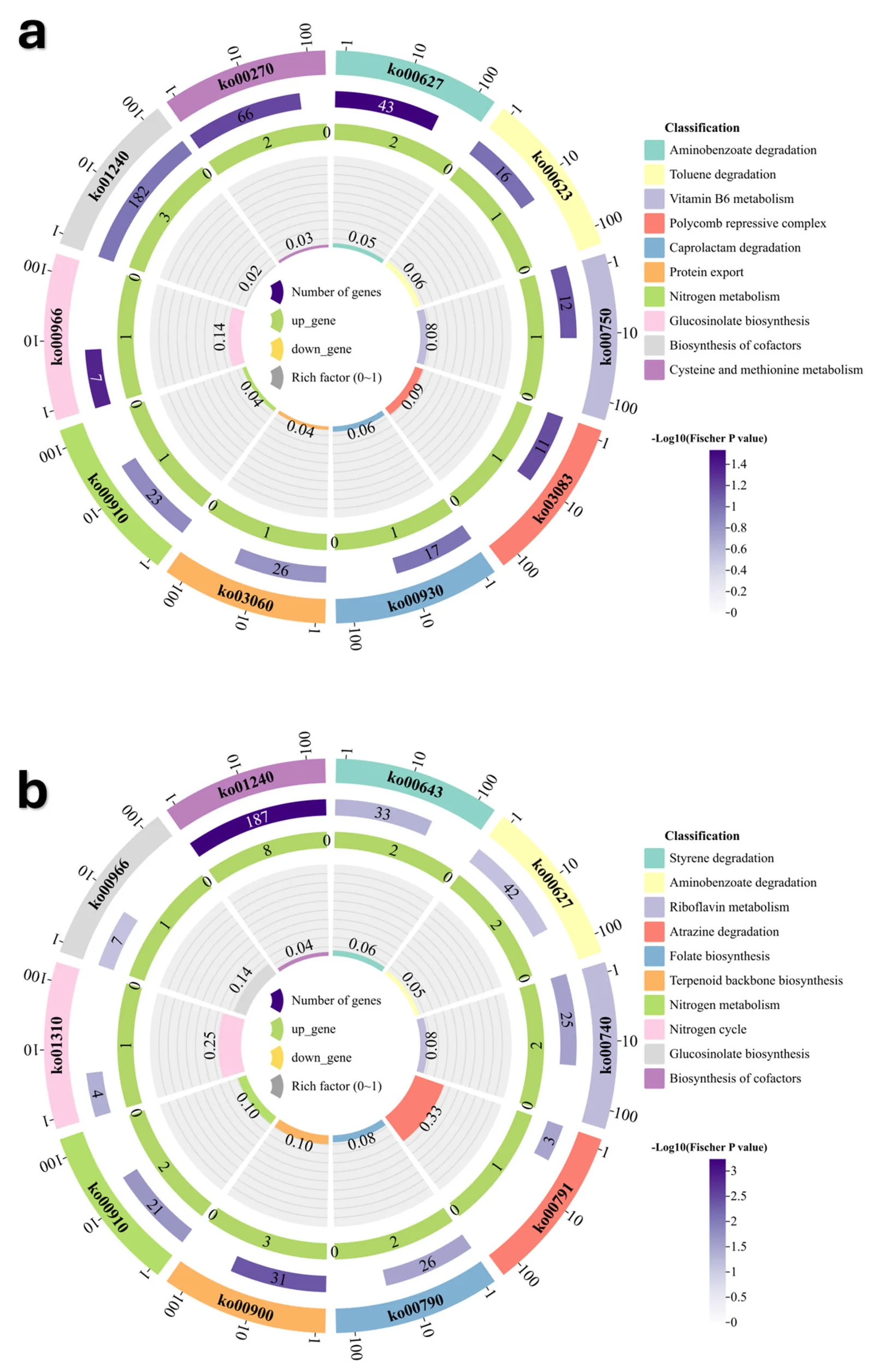
KEGG pathway enrichment of differentially represented genes between the JPY and ZAR clinical cohorts. Circular enrichment plots show results obtained using the: (**a**) ASPNI-centered; and (**b**) *A. welwitschiae*-centered reference projections. The outer-colored sectors identify the KEGG pathways, while the adjacent purple bars show the number of genes assigned to each pathway and are shaded according to −log_10_(Fisher’s *p* value), with darker shading indicating stronger enrichment evidence. The green and yellow tracks report the numbers of JPY-directed and ZAR-directed genes, respectively, based on the JPY–versus–ZAR contrast. The innermost track shows the rich factor, calculated as the proportion of differentially represented genes among all genes assigned to the corresponding pathway. These results describe differences in genomic representation and should not be interpreted as differential gene expression.

Inspection of the differential abundance identified 1218 ASPNI-defined and 1735 Aspwel1-defined features with FDR < 0.05 before applying an effect-size threshold. Requiring both FDR ≤ 0.05 and |log_2_FC| ≥ 1 reduced these sets to 116 ASPNI-defined and 127 Aspwel1-defined features, representing 1.09% and 0.94% of their respective tested feature spaces. Directionality was strongly asymmetric. All 116 ASPNI-defined features showed greater representation in JPY, whereas the Aspwel1-centered analysis identified 125 JPY-directed and only two RSA-directed features (Figure 4). Nevertheless, the genome-wide median log_2_FC remained close to zero under both projections (−0.017 for ASPNI and −0.021 for Aspwel1), indicating that the signal was restricted to a subset of loci rather than reflecting a systematic cohort-wide mapping offset. Several of the largest effects were presence/absence-like. Twenty ASPNI-defined and 14 Aspwel1-defined features had |log_2_FC| > 5. Fifteen of these ASPNI-defined JPY-directed features and nine Aspwel1-defined JPY-directed features had no raw counts in any RSA isolate. For example, ASPNIDRAFT_191617 showed log_2_FC = 14.00 and was detected in seven of eight JPY isolates but none of the RSA isolates. Conversely, BDQ94DRAFT_176242 was absent from all JPY isolates but detected in all eight RSA isolates, producing log_2_FC = −11.09 (FDR = 2.79 × 10^−5^). The second RSA-directed feature, BDQ94DRAFT_179457, had log_2_FC = −6.07 (FDR = 0.029). These extreme effects are consistent with cohort-associated presence/absence or copy-representation differences, although locus structure and reference compatibility may also contribute. Despite the different coordinate systems, 14 KEGG ortholog assignments were significant under both projections. All were JPY-directed; their paired effect sizes were strongly concordant (Pearson *r* = 0.917; median absolute difference = 0.045 log_2_FC). These included the IPI1 pre-rRNA-processing pair, ASPNIDRAFT_53927/BDQ94DRAFT_174822 (log_2_FC = 1.744/1.702), the NADP-dependent alcohol dehydrogenase pair, ASPNIDRAFT_124856/BDQ94DRAFT_185755 (1.437/1.395), the branched-chain amino-acid aminotransferase pair, ASPNIDRAFT_36028/BDQ94DRAFT_183985 (1.415/1.403) and the sterol 24-C-methyltransferase/ERG6 pair, ASPNIDRAFT_42246/BDQ94DRAFT_181604 (1.387/1.324). Additional concordant pairs included protein-S-isoprenylcysteine O-methyltransferase (1.306/1.159), phenol 2-monooxygenase (1.329/1.276), pyridoxamine-5′-phosphate oxidase (1.301/1.348), fumarylacetoacetase (1.070/1.055) and chitosanase (1.045/1.028). These reference-robust signals involved sterol modification, amino-acid and cofactor metabolism, redox and xenobiotic processing, cell-wall turnover and ribosome maturation. Reference-dependent signals were also evident. The ASPNI projection uniquely highlighted nitrate reductase, chitinase, glutathione S-transferase and geranylgeranyl diphosphate synthase among its pathway-mapped differential features. In contrast, the Aspwel1 projection uniquely identified carboxylesterase and a cytochrome P450–NADPH-cytochrome P450 reductase assignment. These one-sided signals may reflect genuine locus differences, but their absence from the reciprocal projection indicates that they should be regarded as reference-contingent until confirmed through direct orthology and locus-level inspection. Pathway mapping linked 31 of the 116 ASPNI-defined and 19 of the 127 Aspwel1-defined differential features to KEGG pathways. Fisher testing identified no pathway that remained significant after FDR correction under either reference. In the ASPNI projection (Figure 5, the strongest unadjusted results were biosynthesis of cofactors (*p* = 5.79 × 10^−4^, FDR = 0.187)) and terpenoid backbone biosynthesis (*p* = 0.0041, FDR = 0.665). Under Aspwel1, aminobenzoate degradation (*p* = 0.029) and glucosinolate biosynthesis (*p* = 0.043) were nominally significant, but both had FDR = 1.0. No nominally significant Fisher pathway was shared between the two projections, demonstrating that threshold over-representation results were sensitive to the selected reference. To further assess the support for the functional assignments explicitly highlighted above, the nine named reference-concordant KO assignments were examined against the corresponding eggNOG outputs and the primary experimental literature (Table 2). Exact eggNOG matches were recovered for seven of the nine ASPNI loci and for two corresponding Aspwel1 loci. Where recovered, ASPNI annotation E values ranged from 1.93 × 10^−11^ to 1.31 × 10^−290^, while the recovered Aspwel1 matches ranged from 4.53 × 10^−27^ to 5.91 × 10^−123^. Loci not recovered as exact identifiers in the corresponding eggNOG export were recorded as NR and were not interpreted as evidence against the KEGG assignment. Independent experimental evidence was identified for all nine functional classes. This included fungal genetic or biochemical evidence supporting IPI1-associated ribosome maturation, branched-chain amino acid transamination, ERG6 sterol C24 methyltransferase activity, STE14-related isoprenylcysteine methylation, phenol hydroxylase activity and fumarylacetoacetase function. The remaining assignments were supported by biochemical, structural or functional characterization of orthologous proteins in other organisms. Collectively, these observations provide independent support for the molecular function annotations assigned to the highlighted reference-concordant loci. They do not demonstrate altered enzyme activity, pathway flux or phenotype in the JPY isolates.

## 4. Discussion

The central finding of this study is that reciprocal projection separated a reproducible cohort-associated component from a larger reference-conditioned component in closely related section-Nigri genomes. Read-level taxonomic classification alone did not cleanly distinguish the public *A. welwitschiae*-labeled libraries from the *A. niger* and South African section-Nigri cohorts: *A. niger*- and *A. welwitschiae*-assigned reads co-occurred in every group, and several Awel libraries contained a larger *A. niger*-assigned component. This ambiguity is consistent with the known difficulty of resolving the *A. niger*/*A. welwitschiae* boundary using limited markers and with phylogenomic evidence that public *Aspergillus* genome labels include non-trivial misidentification [5,46]. The mixed Kaiju profiles should, therefore, not be read as evidence that individual libraries contained two biological species. Rather, they show that protein-level read classification has limited discriminatory power across a closely related species complex and justify the use of whole-genome, reference-aware comparisons. Kaiju assignments were not used to reassign or exclude any samples.

Against this taxonomically ambiguous background, the Awel–versus–ZAR_Nigri analysis produced a small but strongly directional locus-level signal under both coordinate systems. Unsupervised, only 0.41% of tested ASPNI features and 0.44% of tested Aspwel1 features met the joint FDR and effect-size criterion, while the genome-wide median log2 fold change remained near zero. The result is, therefore, inconsistent with a simple genome-wide coverage shift. Moreover, 20 reciprocal feature pairs retained the same Awel-directed pattern across references, supporting a reference-robust core of genomic differentiation. This is biologically plausible in light of extensive inter- and intra-specific diversity reported across section Nigri, including variation in gene content, regulatory proteins and secondary-metabolite clusters [1], as well as the three genomic clades and substantial sequence variation described within *A. niger* sensu stricto [6]. Nevertheless, reciprocal projection is not a phylogenomic species test. The present data support differentiation of the operational Awel cohort from the South African clinical cohort, but definitive reassignment of individual isolates would require genome-wide phylogeny against type or authenticated reference strains. The high-effect tail illustrates why that distinction matters. The Aspwel1-centered analysis contained a compact four-gene block within a 9.8-kb interval, whereas the ASPNI-centered analysis contained one strongly ZAR_Nigri-directed feature and a less pronounced positive tail. Regional clustering is compatible with a shared structural event, differential gene presence, or local copy representation; however, divergent sequence and annotation-specific mappability can produce a similar count pattern. Single-reference short-read alignment is inherently biased toward alleles and regions represented by the selected coordinate system, particularly as divergence increases [7]. The larger Aspwel1 feature catalogue and the predominance of uncharacterized proteins among its significant loci further limit one-to-one functional interpretation. Accordingly, the unmatched high-effect loci are best considered as candidates for targeted locus reconstruction rather than as confirmed gains, losses or copy-number changes.

Functional aggregation was substantially weaker than the locus-level evidence. No Awel–versus–ZAR_Nigri pathway survived Fisher FDR correction, although vitamin B6 metabolism and biosynthesis of cofactors recurred nominally under both projections. Similarly, concordant GSEA ranking of glyoxylate and dicarboxylate metabolism, peroxisome function, cofactor biosynthesis and branched-chain amino-acid biosynthesis provides a useful description of whole-list direction but does not establish pathway alteration. The absence of a single dominant program is consistent with the heterogeneous annotations of the differential loci and with the finding that most statistically resolved effects were modest. More broadly, comparative genomics has shown that metabolic and secondary-metabolite capacities can vary extensively among section-Nigri species and strains [1,17]. In the present study, however, the pathway results are hypothesis-generating because neither enzyme activity nor metabolite abundance was measured, and several generic KEGG labels are derived from ortholog sets that do not imply the named metazoan physiology in fungi. The clinical JPY–versus–ZAR comparison revealed a broader but still localized signal. After joint statistical and effect-size filtering, 116 ASPNI-defined and 127 Aspwel1-defined features were differential, representing approximately 1% of each tested feature space. Directionality was overwhelmingly JPY-associated; however, the near-zero genome-wide medians again argue against a uniform mapping or library-size effect. Most importantly, 14 KEGG ortholog assignments were recovered under both projections with closely matched effect sizes. This reciprocal agreement supports a cohort-associated component involving sterol modification, protein prenylation processing, amino-acid and cofactor metabolism, redox or aromatic-compound processing, cell-wall turnover and ribosome maturation. Fungal pan-genome studies in *Aspergillus* have likewise shown that substantial lineage-specific gene content can remain invisible to a single reference and that accessory variation may contribute to population structure [24,47]. The present contrast is, therefore, compatible with cohort or lineage-associated differentiation between the Japanese and South African clinical collections. Because geography, ancestry, study source and sequencing history were not independently balanced, however, these factors cannot be disentangled, and the result should not be interpreted as a direct effect of country of origin.

Several reciprocal clinical-cohort candidates have clear biological context but require especially restrained interpretation. Sterol 24-C-methyltransferase (ERG6) participates in ergosterol biosynthesis; experimental work in *Aspergillus* fumigatus has shown that Erg6 is essential for viability and affects membrane sterol biology [35]. Protein-S-isoprenylcysteine O-methyltransferase acts downstream in prenylated-protein processing, while geranylgeranyl diphosphate synthase supplies isoprenoid precursors relevant to protein prenylation and other branches of terpenoid metabolism. These observations make the sterol and terpenoid-related signals biologically coherent targets for follow-up. They do not, however, establish altered pathway flux, antifungal susceptibility or virulence. In particular, terpenoid backbone biosynthesis was only nominally enriched in the ASPNI projection and was not reproduced by Fisher testing under Aspwel1. ERG6 was a reproducible locus-level signal, whereas geranylgeranyl diphosphate synthase was reference-contingent. The defensible inference is, therefore, that isoprenoid- and sterol-associated genomic representation differed at selected loci, not that the entire pathway or an antifungal-resistance phenotype differed between cohorts.

A similar distinction applies to chitosanase, chitinase, glutathione S-transferase, phenol 2-monooxygenase, alcohol dehydrogenase and cytochrome P450-related annotations. Together, these functions are compatible with variation in cell-wall remodeling, redox balance and xenobiotic or aromatic-compound metabolism. Chitin and chitosan are dynamic fungal cell-wall components, and their remodeling can influence cell-wall architecture and stress responses [48]. However, only chitosanase was among the reciprocal KO-level clinical signals; chitinase, glutathione S-transferase and geranylgeranyl diphosphate synthase were unique to the ASPNI projection, while the P450-reductase assignment was unique to Aspwel1. Reference-specific recovery weakens any claim of coordinated adaptation. These loci are, therefore, best prioritized for orthology confirmation and phenotypic testing rather than combined into a proposed virulence or detoxification program.

The targeted evidence assessment provides an additional level of support for the molecular functions assigned to the reference concordant loci. Importantly, several assignments are supported by experimental studies in fungal systems rather than by database annotation alone. IPI1-associated ribosome maturation has been demonstrated experimentally in yeast [32], while mitochondrial and cytosolic branched-chain amino acid transaminases have likewise been characterized in yeast [34]. ERG6 has direct functional support in *Aspergillus* species [35], together with biochemical characterization of fungal sterol C24 methyltransferases [36,37]. The STE14 assignment is supported directly by demonstration of farnesylcysteine carboxyl methyltransferase activity in yeast [38], with additional experimental evidence for the biological role of isoprenylcysteine methylation [39,40]. Fungal phenol hydroxylase has been functionally expressed and characterized [41], while genetic interrogation of *fahA* in *Aspergillus nidulans* supports the fumarylacetoacetase assignment [44]. The pyridoxamine 5′ phosphate oxidase assignment is further supported by biochemical and structural characterization of the corresponding enzyme class [42,43]; experimental characterization supports the chitosanase assignment [45]. The NADP-dependent oxidoreductase assignment is similarly consistent with experimentally characterized members of the aldo-keto reductase family [33]. This convergence between reciprocal genomic recovery, sequence-based annotation and independent experimental studies in the literature increases confidence that the highlighted KO assignments represent biologically credible molecular functions rather than unsupported pathway labels. Nevertheless, the experimental studies cited in Table 2 characterize orthologous genes or proteins and do not constitute functional assays of the present clinical isolates. The evidence, therefore, strengthens annotation-level interpretation of the genomic signals but does not establish increased enzyme activity, coordinated pathway activation, altered virulence or antifungal resistance in the JPY cohort.

The study has several limitations that define the next analytical step. The cohorts were modest in size and assembled from different studies, with heterogeneous depth and duplicate representation; robust normalization and replicate-based modeling reduce but cannot eliminate all study-associated confounding. Gene-span counts also combine effects of gene presence, dosage, sequence divergence, annotation boundaries and local mappability. The analysis did not perform formal copy-number segmentation, de novo assembly, structural-variant calling or population-structure adjustment; the 31-mer correspondence analysis establishes sequence similarity rather than complete orthology or conserved locus structure. Finally, pathway interpretation was limited by incomplete annotation and the absence of FDR-supported enrichment. Validation should, therefore, combine long-read or hybrid assemblies, synteny-aware orthology, explicit copy-number and structural-variant analysis and a genome-wide phylogeny incorporating authenticated *A. niger* and *A. welwitschiae* references. Future work could additionally compare the reciprocal whole-genome projections with conventional marker-based approaches, including calmodulin (CaM) sequencing or multilocus sequence typing, to evaluate the extent to which marker-level relationships reproduce the genome-wide cohort structure observed here. For the clinical contrast, antifungal susceptibility, sterol profiling and targeted metabolomics would be needed before linking the highlighted loci to a phenotype. Within these limits, reciprocal projection provides a useful conservative filter: signals reproduced across both references can be prioritized, while reference-specific signals remain candidates requiring direct genomic validation.

## 5. Conclusions

Taken together, the two analyses support a reproducible excess of Awel-associated genomic representation while also demonstrating that the detailed high-effect landscape depends on the reference coordinate system. Aspwel1 tested 2813 more features than ASPNI (26.5% more) and returned 15 more effect-qualified features, but the proportions of the tested feature spaces classified as differential were nearly identical (0.41% and 0.44%). Consequently, the larger raw number of Aspwel1-centric hits should not be interpreted as evidence of greater biological divergence by itself. The shared direction and closely matched reciprocal feature pairs support a stable cohort-associated component, whereas the compact Aspwel1 high-effect block and the single ASPNI-centric RSA-associated feature highlight sensitivity to genome structure, gene modeling, and read mappability. Collectively, reciprocal projection revealed a small and strongly directional locus-level difference between the operational cohorts, while demonstrating substantially weaker functional aggregation. The near-identical proportions of FDR-supported and joint-cutoff loci under the two references argue that the broad cohort signal was not created by a single coordinate system. In contrast, the identities, effect magnitudes and nominal pathway rankings remained reference-conditioned. These differences represent genomic read representation and may reflect dosage, presence–absence variation, divergence, mappability or reference congruence; they do not independently establish transcriptional regulation or formal copy-number states. Overall, the clinical JPY–RSA comparison revealed a localized and overwhelmingly JPY-directed differential-representation signature that was reproducible across the ASPNI and Aspwel1 projections. The 14 concordant KO-level signals, therefore, represent the most reference-robust functional component of the clinical comparison. Targeted evidence assessment showed that the nine concordant functional assignments explicitly highlighted in the Results are supported by sequence-based annotation and independent experimental characterization of the corresponding molecular functions, including several studies conducted in fungal systems. This convergence strengthens confidence in the functional identities assigned to these loci while preserving an important distinction between annotation support and phenotypic validation. Because the present analysis is based on WGS-derived gene-associated read representation, the observed differences may reflect gene presence, dosage, sequence divergence, locus structure or mappability rather than altered gene expression or protein activity. Direct transcriptomic, biochemical, antifungal susceptibility or virulence testing would, therefore, be required to establish the downstream biological consequences of these genomic differences. Extreme presence or absence-like and reference-specific loci additionally require direct locus-level validation. The findings, therefore, support cohort- or lineage-associated genomic differentiation among clinical section *Nigri* isolates but do not independently establish altered transcription, pathway activity, virulence or antifungal resistance.

## Figures and Tables

**Table 1:** Sample metadata and provenance of whole-genome sequencing reads included in the comparative analysis.

Cohort	*n*	Sample/Accession Identifiers	Source of Reads	Associated Publication(s)
ZAR_Nigri section	8	S10, S12, S13, S18, S21, S25, S30, S33	Paired-end WGS reads generated in the present study from clinical *Aspergillus niger* complex isolates collected in Durban, South Africa. Identifiers are internal isolate codes rather than public SRA accessions. Bioproject PRJNA1475450.	Present study
JPY	8	DRR385067, DRR385068, DRR385069, DRR385070, DRR385071, DRR385072, DRR385075, DRR385076	Paired-end WGS reads from eight human clinical *A. niger* isolates collected in Japan. BioProject PRJDB13764.	[4]
USA	8	SRR33239775, SRR33239776, SRR33239779, SRR33239784, SRR33239789, SRR33239794, SRR33239795, SRR33239812	WGS reads from human-associated clinical black-*Aspergillus* isolates collected in California. BioProject PRJNA1253755.	[9]
Awel	7	SRR10244387, SRR10716349, SRR19542939, SRR23190057, SRR24957354, SRR6793083, SRR8399229	Multiple sources, exhaustive of the *A. welwitschiae* available data in NCBI	[1,3,5,10].

**Table 2:** Experimental support for reference-based functional annotations highlighted in Section 3.5.

KO	Reciprocal Loci	Functional Assignment	eggNOG E-Value (ASPNI/Aspwel1)	Experimental Support	Interpretation
K14827	ASPNIDRAFT_53927 BDQ94DRAFT_174822	Pre-rRNA-processing protein IPI1	0/0	Yeast *Ipi1*-containing complex shown to participate in late pre-rRNA processing and 60S-subunit maturation/export [32].	Strong fungal support for the IPI1/ribosome-biogenesis annotation; altered ribosome activity in JPY was not tested.
K00002	ASPNIDRAFT_124856 BDQ94DRAFT_185755	NADP-dependent alcohol dehydrogenase	1.31 × 10^−290^/0	NADPH-dependent aldo-keto reductase/oxidoreductase family characterized by cDNA and deduced protein-sequence analysis [33].	Very strong sequence support with moderate ortholog-level functional support; the experimental source is non-fungal.
K00826	ASPNIDRAFT_36028 BDQ94DRAFT_183985	Branched-chain amino-acid aminotransferase	1.32 × 10^−276^/0	Mitochondrial and cytosolic branched-chain amino-acid transaminases experimentally characterized in yeast [34].	Strong fungal support for EC 2.6.1.42; altered amino-acid flux in the study isolates was not measured.
K00559	ASPNIDRAFT_42246 BDQ94DRAFT_181604	Sterol 24-C-methyltransferase (ERG6)	0/0	ERG6 function established in *Aspergillus* [35], with complementary mutational/biochemical [36] and purified-enzyme evidence from fungi [37].	Very strong fungal genetic and biochemical support for ERG6 identity; sterol abundance and antifungal susceptibility were not measured.
K00587	ASPNIDRAFT_186640 BDQ94DRAFT_129302	Protein-S-isoprenylcysteine O-methyltransferase (STE14-like)	3.12 × 10^−178^/0	Yeast STE14 shown to encode farnesylcysteine carboxyl methyltransferase [38], with independent functional support for isoprenylcysteine methylation [39,40].	Very strong fungal ortholog support for isoprenylcysteine methyltransferase function; altered prenylation was not tested.
K03380	ASPNIDRAFT_38796 BDQ94DRAFT_167524	Phenol 2-monooxygenase/phenol hydroxylase	1.86 × 10^−12^/4.53 × 10^−27^	Fungal phenol hydroxylase cloned from *Trichosporon cutaneum* and functionally expressed in *Escherichia coli* [41].	Strong fungal experimental support; reciprocal eggNOG matches support the annotation, not increased aromatic-compound metabolism.
K00275	ASPNIDRAFT_201497 BDQ94DRAFT_166391	Pyridoxamine-5′-phosphate oxidase	7.65 × 10^−183^/5.91 × 10^−123^	Recombinant pyridoxine-5′-phosphate oxidase characterized biochemically [42] and structurally/mechanistically [43].	Strong biochemical/structural support with strong reciprocal sequence support; altered vitamin-B6 metabolism was not measured.
K01555	ASPNIDRAFT_37286 BDQ94DRAFT_178484	Fumarylacetoacetase	1.92 × 10^−65^/0	Fungal tyrosine-degradation pathway experimentally interrogated in *Aspergillus nidulans*, including direct genetic analysis of *fahA* [44].	Very strong fungal genetic support for the fumarylacetoacetase assignment; altered tyrosine catabolism in JPY was not tested.
K01233	ASPNIDRAFT_190824 BDQ94DRAFT_165368	Chitosanase	1.93 × 10^−11^/0	*Bacillus subtilis* csn shown experimentally to encode chitosanase, followed by enzyme characterization [45].	Strong direct enzymatic support from a bacterial ortholog; altered fungal cell-wall remodeling was not demonstrated.

## Data Availability

The whole-genome sequencing data generated in this study for seven South African clinical *Aspergillus nigri* section isolates are available in the NCBI Sequence Read Archive (SRA) under BioProject accession PRJNA1475450 and are available in the following reviewer link: https://dataview.ncbi.nlm.nih.gov/object/PRJNA1475450?reviewer=ccf74rro4n7d9tkb6vv5dgokr2 (accessed on 30 August 2026). Comparative publicly available sequencing datasets were retrieved from the NCBI SRA and comprised 26 additional datasets from Nigeria (*n* = 9; BioProject PRJNA1265142), Japan (*n* = 10; PRJDB16281, *n* = 9; PRJNA743902, *n* = 1), the United States (*n* = 3; PRJNA743902, PRJNA807647, and SRP151231) and the International Space Station (*n* = 4; PRJNA807647, *n* = 1; and PRJNA667181, *n* = 3).

## Supplementary Materials

The following supporting information can be downloaded at: https://www.mdpi.com/article/10.3390/pathogens15090941/s1, File S1: Supporting data.

## Abbreviations

The following abbreviations are used in this manuscript:

WGS	Whole-genome sequencing
RLE	Relative log expression
GLM	Generalized linear model
FDR	False discovery rate
GFF3	General Feature Format, version 3
GO	Gene ontology
GOA	Gene ontology annotation
GSEA	Gene-set enrichment analysis
GTF	Gene-transfer Format
NES	Normalized enrichment score
KEGG	Kyoto Encyclopedia of Genes and Genomes
KO	KEGG Orthology
log_2_FC	Log_2_ fold change

## Appendix A

**Table A1:** Mapping quality—reference-based alignment results of samples.

*A. niger* Projection							
Sample	Total Alignments	Mapped (%)	Supplementary (%)	Unmapped (%)	Duplication Rate (%)	Mean Coverage (×)	Mean MAPQ
DRR385076_JPY	11,514,284	85.617	0.875	14.383	22.94	55.804	58.819
S10_ZAR	7,452,237	89.397	0.173	10.603	11.38	28.127	58.904
S12_ZAR	4,986,004	88.931	0.196	11.069	8.62	18.630	58.995
S13_ZAR	11,543,790	90.841	0.178	9.159	16.64	44.217	59.085
S18_ZAR	14,634,249	90.973	0.149	9.027	20.12	56.023	59.023
S21_ZAR	17,419,055	90.866	0.155	9.134	23.19	66.848	59.014
S25_ZAR	5,028,982	91.384	0.201	8.616	13.18	19.297	59.052
S30_ZAR	7,311,890	89.795	0.177	10.205	11.67	27.625	59.054
S33_ZAR	3,951,808	88.861	0.190	11.139	7.00	14.799	59.041
SRR10244387_Awel	14,995,813	89.423	0.167	10.577	21.75	52.248	58.881
SRR10716349_Awel	43,982,014	87.880	0.262	12.120	47.82	160.077	58.718
SRR19542939_Awel	25,670,055	88.314	0.178	11.686	42.18	94.910	59.103
SRR23190057_Awel	30,454,456	85.203	0.186	14.797	46.48	104.320	58.865
SRR24957354_Awel	107,824,852	86.658	0.796	13.342	77.89	337.362	58.862
SRR33239775_USA	61,047,654	94.721	0.107	5.279	69.81	158.712	59.110
SRR33239776_USA	54,390,068	91.482	0.073	8.518	68.67	139.247	59.022
SRR33239779_USA	59,550,795	90.844	0.122	9.156	69.47	149.405	59.040
SRR33239784_USA	52,454,924	95.503	0.094	4.497	68.19	139.833	58.953
SRR33239789_USA	40,762,201	92.509	0.227	7.491	64.14	104.400	58.947
SRR33239794_USA	19,276,437	85.260	0.162	14.740	24.19	66.813	58.740
SRR33239795_USA	78,638,373	91.683	0.078	8.317	74.27	194.435	59.055
SRR33239812_USA	23,841,625	91.759	0.099	8.241	29.79	90.441	59.209
SRR6793083_Awel	32,236,473	86.174	0.252	13.826	37.48	106.994	58.643
SRR8399229_Awel	54,829,726	85.037	0.244	14.963	53.84	194.104	58.884

**Table A2:** Mapping quality—reference-based alignment results of samples.

*A. welwitschiae* Projection							
Sample	Total Alignments	Mapped (%)	Supplementary (%)	Unmapped (%)	Duplication Rate (%)	Mean Coverage (×)	Mean MAPQ
DRR385067_JPY	25,176,563	89.941	1.048	10.059	36.42	56.417	53.229
DRR385068_JPY	24,169,818	88.680	1.171	11.320	34.63	53.598	53.560
DRR385069_JPY	18,530,445	87.851	0.865	12.149	30.63	40.287	49.136
DRR385070_JPY	24,162,260	87.925	0.907	12.075	35.31	53.127	49.521
DRR385071_JPY	21,019,485	85.468	0.951	14.532	31.71	44.415	51.537
DRR385072_JPY	14,960,476	88.662	1.441	11.338	34.37	61.821	51.519
DRR385075_JPY	6,658,720	87.768	1.592	12.232	14.97	33.514	52.449
DRR385076_JPY	11,550,322	88.843	1.185	11.157	22.91	54.076	52.385
S10_ZAR	7,457,710	92.282	0.246	7.718	11.04	27.077	53.240
S12_ZAR	4,990,872	92.484	0.293	7.516	8.21	18.070	52.906
S13_ZAR	11,550,156	93.249	0.233	6.751	16.45	42.314	52.868
S18_ZAR	14,642,040	92.740	0.203	7.260	19.93	53.219	53.194
S21_ZAR	17,430,620	92.785	0.221	7.215	23.01	63.623	53.354
S25_ZAR	5,031,393	93.728	0.249	6.272	12.79	18.440	51.899
S30_ZAR	7,317,177	92.470	0.249	7.530	11.43	26.529	53.269
S33_ZAR	3,954,330	92.252	0.254	7.748	6.56	14.329	53.282
SRR10244387_Awel	15,002,859	91.088	0.214	8.912	21.54	49.549	49.890
SRR10716349_Awel	44,018,226	90.461	0.344	9.539	47.79	153.422	52.291
SRR19542939_Awel	25,703,425	89.622	0.308	10.378	42.35	89.363	50.156
SRR23190057_Awel	30,495,874	89.802	0.322	10.198	46.63	102.747	53.480
SRR24957354_Awel	108,081,340	90.675	1.031	9.325	77.90	330.521	54.693
SRR33239775_USA	61,058,641	90.871	0.125	9.129	69.88	140.441	50.569
SRR33239776_USA	54,399,452	92.486	0.090	7.514	68.52	131.030	53.016
SRR33239779_USA	59,563,740	93.020	0.144	6.980	69.30	142.458	53.366
SRR33239784_USA	52,460,393	92.362	0.105	7.638	68.23	125.115	44.764
SRR33239789_USA	40,771,155	92.090	0.249	7.910	63.97	96.678	50.520
SRR33239794_USA	19,290,975	86.222	0.238	13.778	24.11	62.363	48.382
SRR33239795_USA	78,645,979	92.557	0.087	7.443	74.11	182.642	53.288
SRR33239812_USA	23,884,132	88.711	0.276	11.289	30.02	80.260	51.281
SRR6793083_Awel	32,289,180	88.517	0.415	11.483	37.69	101.535	49.591
SRR8399229_Awel	54,763,048	94.006	0.122	5.994	53.91	202.889	58.712

## References

[1] Vesth T.C., Nybo J.L., Theobald S., Frisvad J.C., Larsen T.O., Nielsen K.F., Hoof J.B., Brandl J., Salamov A., Riley R. (2018). Investigation of inter-and intraspecies variation through genome sequencing of Aspergillus section Nigri. Nat. Genet..

[2] De Vries R.P., Riley R., Wiebenga A., Aguilar-Osorio G., Amillis S., Uchima C.A., Anderluh G., Asadollahi M., Askin M., Barry K. (2017). Comparative genomics reveals high biological diversity and specific adaptations in the industrially and medically important fungal genus Aspergillus. Genome Biol..

[3] Nargesi S., Jafarzadeh J., Najafzadeh M.J., Nouripour-Sisakht S., Haghani I., Abastabar M., Ilkit M., Hedayati M.T. (2022). Molecular identification and antifungal susceptibility of clinically relevant and cryptic species of Aspergillus sections Flavi and Nigri. J. Med. Microbiol..

[4] Bian C., Kusuya Y., Sklenář F., D’Hooge E., Yaguchi T., Ban S., Visagie C.M., Houbraken J., Takahashi H., Hubka V. (2022). Reducing the number of accepted species in Aspergillus series Nigri. Stud. Mycol..

[5] Steenwyk Jacob L., Balamurugan C., Huzefa A.R., Gonçalves C., Li N., Martin F., Berman J., Nicholas H.O., John G.G., Gustavo H.G. (2024). Phylogenomics reveals extensive misidentification of fungal strains from the genus Aspergillus. Microbiol. Spectr..

[6] Seekles S.J., Punt M., Savelkoel N., Houbraken J., Wösten H.A.B., Ohm R.A., Ram A.F.J. (2022). Genome sequences of 24 Aspergillus niger sensu stricto strains to study strain diversity, heterokaryon compatibility, and sexual reproduction. G3.

[7] Huang L., Popic V., Batzoglou S. (2013). Short read alignment with populations of genomes. Bioinformatics.

[8] Naicker S., Mohanlall V., Ngubane S., Mellem J., McHunu N.P. (2023). Phenotypic array for identification and screening of antifungals against Aspergillus isolates from respiratory infections in KwaZulu Natal, South Africa. J. Fungi.

[9] Wang Y., Aziz M., Bush K., Davis G.S., Foster J.T., Hallstrøm S., Jones A., Keim P.S., Leventis R., Liu C.M. (2025). Triazole Resistance and Misidentification of Aspergillus tubingensis in Southern California. JAMA Netw. Open.

[10] Quintanilha-Peixoto G., Marone M.P., Raya F.T., José J., Oliveira A., Fonseca P.L.C., Tomé L.M.R., Bortolini D.E., Kato R.B., Araújo D.S. (2022). Phylogenomics and gene selection in Aspergillus welwitschiae: Possible implications in the pathogenicity in Agave sisalana. Genomics.

[11] Andrews S. (2010). FastQC: A Quality Control Tool for High Throughput Sequence Data.

[12] Ohta T., Nakazato T., Bono H. (2017). Calculating the quality of public high-throughput sequencing data to obtain a suitable subset for reanalysis from the Sequence Read Archive. GigaScience.

[13] Ewels P., Magnusson M., Lundin S., Käller M. (2016). MultiQC: Summarize analysis results for multiple tools and samples in a single report. Bioinformatics.

[14] Bolger A.M., Lohse M., Usadel B. (2014). Trimmomatic: A flexible trimmer for Illumina sequence data. Bioinformatics.

[15] Arkin A.P., Cottingham R.W., Henry C.S., Harris N.L., Stevens R.L., Maslov S., Dehal P., Ware D., Perez F., Canon S. (2018). KBase: The United States department of energy systems biology knowledgebase. Nat. Biotechnol..

[16] Menzel P., Ng K.L., Krogh A. (2016). Fast and sensitive taxonomic classification for metagenomics with Kaiju. Nat. Commun..

[17] Andersen M.R., Salazar M.P., Schaap P.J., van de Vondervoort P.J.I., Culley D., Thykaer J., Frisvad J.C., Nielsen K.F., Albang R., Albermann K. (2011). Comparative genomics of citric-acid-producing Aspergillus niger ATCC 1015 versus enzyme-producing CBS 513.88. Genome Res..

[18] Li H. (2013). Aligning sequence reads, clone sequences and assembly contigs with BWA-MEM. arXiv.

[19] Danecek P., Bonfield J.K., Liddle J., Marshall J., Ohan V., Pollard M.O., Whitwham A., Keane T., McCarthy S.A., Davies R.M. (2021). Twelve years of SAMtools and BCFtools. GigaScience.

[20] Okonechnikov K., Conesa A., García-Alcalde F. (2016). Qualimap 2: Advanced multi-sample quality control for high-throughput sequencing data. Bioinformatics.

[21] Lawrence M., Huber W., Pagès H., Aboyoun P., Carlson M., Gentleman R., Morgan M.T., Carey V.J. (2013). Software for computing and annotating genomic ranges. PLoS Comput. Biol..

[22] Huber W., Carey V.J., Gentleman R., Anders S., Carlson M., Carvalho B.S., Bravo H.C., Davis S., Gatto L., Girke T. (2015). Orchestrating high-throughput genomic analysis with Bioconductor. Nat. Methods.

[23] Robinson M.D., McCarthy D.J., Smyth G.K. (2010). EdgeR: A Bioconductor package for differential expression analysis of digital gene expression data. Bioinformatics.

[24] McCarthy C.G.P., Fitzpatrick D.A. (2019). Pan-genome analyses of model fungal species. Microb. Genom..

[25] Benjamini Y., Hochberg Y. (1995). Controlling the false discovery rate: A practical and powerful approach to multiple testing. J. R. Stat. Soc. Ser. B (Methodol.).

[26] Götz S., García-Gómez J.M., Terol J., Williams T.D., Nagaraj S.H., Nueda M.J., Robles M., Talón M., Dopazo J., Conesa A. (2008). High-throughput functional annotation and data mining with the Blast2GO suite. Nucleic Acids Res..

[27] Cantalapiedra C.P., Hernández-Plaza A., Letunic I., Bork P., Huerta-Cepas J. (2021). EggNOG-mapper v2: Functional annotation, orthology assignments, and domain prediction at the metagenomic scale. Mol. Biol. Evol..

[28] Kanehisa M., Goto S. (2000). KEGG: Kyoto encyclopedia of genes and genomes. Nucleic Acids Res..

[29] BioBam Bioinformatics (2026). Combined Pathway Analysis. OmicsBox User Manual.

[30] BioBam Bioinformatics (2026). Gene Set Enrichment Analysis. OmicsBox User Manual.

[31] Subramanian A., Tamayo P., Mootha V.K., Mukherjee S., Ebert B.L., Gillette M.A., Paulovich A., Pomeroy S.L., Golub T.R., Lander E.S. (2005). Gene set enrichment analysis: A knowledge-based approach for interpreting genome-wide expression profiles. Proc. Natl. Acad. Sci. USA.

[32] Galani K., Nissan T.A., Petfalski E., Tollervey D., Hurt E. (2004). Rea1, a dynein-related nuclear AAA-ATPase, is involved in late rRNA processing and nuclear export of 60 S subunits. J. Biol. Chem..

[33] Bohren K.M., Bullock B., Wermuth B., Gabbay K.H. (1989). The aldo-keto reductase superfamily: CDNAs and deduced amino acid sequences of human aldehyde and aldose reductases. J. Biol. Chem..

[34] Kispal G., Steiner H., Rolinski B., Lill R. (1996). Mitochondrial and cytosolic branched-chain amino acid transaminases from yeast, homologs of the myc oncogene-regulated Eca39 protein. J. Biol. Chem..

[35] Xie J., Rybak J.M., Martin-Vicente A., Guruceaga X., Thorn H.I., Nywening A.V., Ge W., Parker J.E., Kelly S.L., Rogers P.D. (2024). The sterol C-24 methyltransferase encoding gene, erg6, is essential for viability of Aspergillus species. Nat. Commun..

[36] Jayasimha P., Nes W.D. (2008). Photoaffinity labeling and mutational analysis of 24-C-sterol methyltransferase defines the AdoMet binding site. Lipids.

[37] Ganapathy K., Kanagasabai R., Nguyen T.T.M., Nes W.D. (2011). Purification, characterization and inhibition of sterol C24-methyltransferase from Candida albicans. Arch. Biochem. Biophys..

[38] Sapperstein S., Berkower C., Michaelis S. (1994). Nucleotide sequence of the yeast STE14 gene, which encodes farnesylcysteine carboxyl methyltransferase, and demonstration of its essential role in a-factor export. Mol. Cell. Biol..

[39] Chen Y., McQuade K.J., Guan X.-J., Thomason P.A., Wert M.S., Stock J.B., Cox E.C. (2007). Isoprenylcysteine carboxy methylation is essential for development in Dictyostelium discoideum. Mol. Biol. Cell.

[40] Huizinga D.H., Omosegbon O., Omery B., Crowell D.N. (2008). Isoprenylcysteine methylation and demethylation regulate abscisic acid signaling in Arabidopsis. Plant Cell.

[41] Kälin M., Neujahr H.Y., Weissmahr R.N., Sejlitz T., Jöhl R., Fiechter A., Reiser J. (1992). Phenol hydroxylase from Trichosporon cutaneum: Gene cloning, sequence analysis, and functional expression in Escherichia coli. J. Bacteriol..

[42] Musayev F.N., Di Salvo M.L., Ko T.P., Schirch V., Safo M.K. (2003). Structure and properties of recombinant human pyridoxine 5′-phosphate oxidase. Protein Sci..

[43] Safo M.K., Musayev F.N., Schirch V. (2005). Structure of Escherichia coli pyridoxine 5′-phosphate oxidase in a tetragonal crystal form: Insights into the mechanistic pathway of the enzyme. Biol. Crystallogr..

[44] Fernández-Cañón J.M., Peñalva M.A. (1995). Fungal metabolic model for human type I hereditary tyrosinaemia. Proc. Natl. Acad. Sci. USA.

[45] Rivas L.A., Parro V.C., Moreno-Paz M., Mellado R.P. (2000). The Bacillus subtilis 168 csn gene encodes a chitosanase with similar properties to a Streptomyces enzyme. Microbiology.

[46] Qi G., Hao L., Gan Y., Xin T., Lou Q., Xu W., Song J. (2024). Identification of closely related species in Aspergillus through Analysis of Whole-Genome. Front. Microbiol..

[47] Lofgren L.A., Ross B.S., Cramer R.A., Stajich J.E. (2022). The pan-genome of Aspergillus fumigatus provides a high-resolution view of its population structure revealing high levels of lineage-specific diversity driven by recombination. PLoS Biol..

[48] Kappel L., Münsterkötter M., Sipos G., Escobar Rodriguez C., Gruber S. (2020). Chitin and chitosan remodeling defines vegetative development and Trichoderma biocontrol. PLoS Pathog..

