# Supplementary material for: Reciprocal Genome Projection Reveals Reference-Conditioned Functional Variation in Clinical Aspergillus Section Nigri Isolates
Source: Pathogens. 2026 Sep 4;15(9):941. doi: 10.3390/pathogens15090941 (PMC13610854; doi:10.3390/pathogens15090941)
Supplement: Supplementary file 1 [file pathogens-15-00941-s001.zip › pathogens-4521853-supplementary File S1.pdf]

ORTHOLOGY: K14827 Help

**Entry**	K14827 KO
**Symbol**	IPI1, TEX10
**Name**	pre-rRNA-processing protein IPI1
**Pathway**	map03083 Polycomb repressive complex
**Brite**	KEGG Orthology (KO) [BR:ko00001]<br>09120 Genetic Information Processing<br>09126 Chromosome<br>03083 Polycomb repressive complex<br>K14827 IPI1, TEX10; pre-rRNA-processing protein IPI1<br>09180 Brite Hierarchies<br>09182 Protein families: genetic information processing<br>03009 Ribosome biogenesis<br>K14827 IPI1, TEX10; pre-rRNA-processing protein IPI1<br>Ribosome biogenesis [BR:ko03009]<br>Eukaryotic type<br>Pre-60S particles<br>5-FMC complex<br>K14827 IPI1, TEX10; pre-rRNA-processing protein IPI1
**Genes**	HSA: 54881(TEX10)<br>PTR: 464618(TEX10)<br>PPS: 100988922(TEX10)<br>GGO: 101135096(TEX10)<br>PON: 100171926(TEX10)<br>PPYG: 129044288(TEX10)<br>NLE: 100594681(TEX10)<br>HMH: 116475331(TEX10)<br>SSYN: 129479101(TEX10)<br>MCC: 714536(TEX10)

	» show all<br>TaxonomyUniProt
**Reference**	PMID:15528184
**Authors**	Galani K, Nissan TA, Petfalski E, Tollervey D, Hurt E
**Title**	Rea1, a dynein-related nuclear AAA-ATPase, is involved in late rRNA processing and nuclear export of 60 S subunits.
**Journal**	J Biol Chem 279:55411-8 (2004)<br>DOI:10.1074/jbc.M406876200
**Sequence**	[sce:YHR085W]

KEGG **ORTHOLOGY: K00002**

Help

**Entry**	K00002 KO
**Symbol**	AKR1A1, adh
**Name**	alcohol dehydrogenase (NADP+) [EC:1.1.1.2]
**Pathway**	map00010 Glycolysis / Gluconeogenesis<br>map00040 Pentose and glucuronate interconversions<br>map00053 Ascorbate and aldarate metabolism<br>map00561 Glycerolipid metabolism<br>map00620 Pyruvate metabolism<br>map00930 Caprolactam degradation<br>map01100 Metabolic pathways<br>map01110 Biosynthesis of secondary metabolites<br>map01120 Microbial metabolism in diverse environments<br>map01220 Degradation of aromatic compounds

	map01240 Biosynthesis of cofactors<br><br>map05208 Chemical carcinogenesis - reactive oxygen species
**Module**	M00014 Glucuronate pathway (uronate pathway)<br><br>M00129 Ascorbate biosynthesis, animals, glucose-1P => ascorbate
**Reaction**	R00746 ethanol:NADP+ oxidoreductase<br><br>R01041 glycerol:NADP+ oxidoreductase<br><br>R01481 L-gulonate:NADP+ 6-oxidoreductase<br><br>R05231 6-hydroxyhexanoate:NADP+ oxidoreductase
**Brite**	KEGG Orthology (KO) [BR:ko00001]<br>09100 Metabolism<br>09101 Carbohydrate metabolism<br>00010 Glycolysis / Gluconeogenesis<br>K00002 AKR1A1, adh; alcohol dehydrogenase (NADP+)<br>00040 Pentose and glucuronate interconversions<br>K00002 AKR1A1, adh; alcohol dehydrogenase (NADP+)<br>00053 Ascorbate and aldarate metabolism<br>K00002 AKR1A1, adh; alcohol dehydrogenase (NADP+)<br>00620 Pyruvate metabolism<br>K00002 AKR1A1, adh; alcohol dehydrogenase (NADP+)<br>09103 Lipid metabolism<br>00561 Glycerolipid metabolism<br>K00002 AKR1A1, adh; alcohol dehydrogenase (NADP+)<br>09111 Xenobiotics biodegradation and metabolism<br>00930 Caprolactam degradation<br>K00002 AKR1A1, adh; alcohol dehydrogenase (NADP+)<br>09160 Human Diseases<br>09161 Cancer: overview<br>05208 Chemical carcinogenesis - reactive oxygen species<br>K00002 AKR1A1, adh; alcohol dehydrogenase (NADP+)<br>09180 Brite Hierarchies<br>09183 Protein families: signaling and cellular processes<br>04147 Exosome<br>K00002 AKR1A1, adh; alcohol dehydrogenase (NADP+)<br>Enzymes [BR:ko01000]<br>1. Oxidoreductases

	1.1 Acting on the CH-OH group of donors<br>1.1.1 With NAD+ or NADP+ as acceptor<br>1.1.1.2 alcohol dehydrogenase (NADP+)<br>K00002 AKR1A1, adh; alcohol dehydrogenase (NADP+)<br>Exosome [BR:ko04147]<br>Exosomal proteins<br>Exosomal proteins of other body fluids (saliva and urine)<br>K00002 AKR1A1, adh; alcohol dehydrogenase (NADP+)
**Other DBs**	COG: COG0656<br><br>GO: 0008106
**Genes**	HSA: 10327(AKR1A1)<br><br>PTR: 741418(AKR1A1)<br><br>PPS: 100990701(AKR1A1)<br><br>GGO: 101124639(AKR1A1)<br><br>PON: 100173796(AKR1A1)<br><br>PPYG: 129037826(AKR1A1)<br><br>NLE: 100581229(AKR1A1)<br><br>HMH: 116473092(AKR1A1)<br><br>SSYN: 129469095(AKR1A1)<br><br>MCC: 693380(AKR1A1)<br><br>» show all<br>TaxonomyUniProt
**Reference**	PMID:2498333
**Authors**	Bohren KM, Bullock B, Wermuth B, Gabbay KH
**Title**	The aldo-keto reductase superfamily. cDNAs and deduced amino acid sequences of human aldehyde and aldose reductases.
**Journal**	J Biol Chem 264:9547-51 (1989)
**Sequence**	[hsa:10327]

ORTHOLOGY: K00826

Help

**Entry**	K00826 KO
**Symbol**	E2.6.1.42, ilvE
**Name**	branched-chain amino acid aminotransferase [EC:2.6.1.42]
**Pathway**	map00270 Cysteine and methionine metabolism<br>map00280 Valine, leucine and isoleucine degradation<br>map00290 Valine, leucine and isoleucine biosynthesis<br>map00770 Pantothenate and CoA biosynthesis<br>map00966 Glucosinolate biosynthesis<br>map01100 Metabolic pathways<br>map01110 Biosynthesis of secondary metabolites<br>map01210 2-Oxocarboxylic acid metabolism<br>map01230 Biosynthesis of amino acids<br>map01240 Biosynthesis of cofactors
**Module**	M00019 Valine/isoleucine biosynthesis, pyruvate => valine / 2-oxobutanoate => isoleucine<br>M00036 Leucine degradation, leucine => acetoacetate + acetyl-CoA<br>M00119 Pantothenate biosynthesis, valine/L-aspartate => pantothenate<br>M00570 Isoleucine biosynthesis, threonine => 2-oxobutanoate => isoleucine
**Reaction**	R01090 L-leucine:2-oxoglutarate aminotransferase<br>R01214 L-valine:2-oxoglutarate aminotransferase<br>R02199 L-isoleucine:2-oxoglutarate aminotransferase<br>R08622 homomethionine:2-oxo-acid aminotransferase<br>R08626 dihomomethionine:2-oxo-acid aminotransferase<br>R10991 2-aminobutanoate:2-oxoglutarate aminotransferase

**Disease**	H02546 Hypervalinemia and hyperleucine-isoleucinemia
**Brite**	KEGG Orthology (KO) [BR:ko00001]<br>09100 Metabolism<br>09105 Amino acid metabolism<br>00270 Cysteine and methionine metabolism<br>K00826 E2.6.1.42, ilvE; branched-chain amino acid aminotransferase<br>00280 Valine, leucine and isoleucine degradation<br>K00826 E2.6.1.42, ilvE; branched-chain amino acid aminotransferase<br>00290 Valine, leucine and isoleucine biosynthesis<br>K00826 E2.6.1.42, ilvE; branched-chain amino acid aminotransferase<br>09108 Metabolism of cofactors and vitamins<br>00770 Pantothenate and CoA biosynthesis<br>K00826 E2.6.1.42, ilvE; branched-chain amino acid aminotransferase<br>09110 Biosynthesis of other secondary metabolites<br>00966 Glucosinolate biosynthesis<br>K00826 E2.6.1.42, ilvE; branched-chain amino acid aminotransferase<br>09180 Brite Hierarchies<br>09181 Protein families: metabolism<br>01007 Amino acid related enzymes<br>K00826 E2.6.1.42, ilvE; branched-chain amino acid aminotransferase<br>Enzymes [BR:ko01000]<br>2. Transferases<br>2.6 Transferring nitrogenous groups<br>2.6.1 Transaminases<br>2.6.1.42 branched-chain-amino-acid transaminase<br>K00826 E2.6.1.42, ilvE; branched-chain amino acid aminotransferase<br>Amino acid related enzymes [BR:ko01007]<br>Aminotransferase (transaminase)<br>Class IV<br>K00826 E2.6.1.42, ilvE; branched-chain amino acid aminotransferase
**Other DBs**	COG: COG0115<br><br>GO: 0004084
**Genes**	HSA: 586(BCAT1) 587(BCAT2)<br><br>PTR: 456189(BCAT2) 465345(BCAT1)<br><br>PPS: 100994347(BCAT1) 100995385(BCAT2)

	GGO: 101141824(BCAT1) 129528628(BCAT2)<br><br>PON: 100171664(BCAT2) 100460550(BCAT1)<br><br>PPYG: 129009712(BCAT1) 129021132(BCAT2)<br><br>NLE: 100591100(BCAT1) 100591118(BCAT2)<br><br>HMH: 116472780(BCAT1) 116478232(BCAT2)<br><br>SSYN: 129459288(BCAT2) 129483244(BCAT1)<br><br>MCC: 707321(BCAT1) 718305(BCAT2)

KEGG **ORTHOLOGY: K00559**

Help

**Entry**	K00559 KO
**Symbol**	SMT1, ERG6
**Name**	sterol 24-C-methyltransferase [EC:2.1.1.41]
**Pathway**	map00100 Steroid biosynthesis<br><br>map01100 Metabolic pathways<br><br>map01110 Biosynthesis of secondary metabolites
**Module**	M00102 Ergocalciferol biosynthesis, FPP => ergosterol/ergocalciferol<br><br>M00917 Phytosterol biosynthesis, squalene 2,3-epoxide => campesterol/sitosterol
**Reaction**	R04427 S-adenosyl-L-methionine:zymosterol C-methyltransferase<br><br>R07481
**Brite**	KEGG Orthology (KO) [BR:ko00001]<br>09100 Metabolism<br>09103 Lipid metabolism<br>00100 Steroid biosynthesis<br>K00559 SMT1, ERG6; sterol 24-C-methyltransferase<br>Enzymes [BR:ko01000]

	2. Transferases<br>2.1 Transferring one-carbon groups<br>2.1.1 Methyltransferases<br>2.1.1.41 sterol 24-C-methyltransferase<br>K00559 SMT1, ERG6; sterol 24-C-methyltransferase
**Other DBs**	COG: COG0500<br><br>GO: 0003838
**Genes**	NAI: NECAME_03546<br><br>AQU: 100639491<br><br>CCLM: 134182813 134183533 134183534<br><br>OSA: 4342690(Smt1-1) 9269350<br><br>DOSA: Os03g0807600 Os07g0206700<br><br>OBR: 102713236<br><br>OGL: 127755018 127766216 127779788<br><br>BDI: 100826864 100834229<br><br>ATS: 109734415 109756778<br><br>TDC: 119291992 119304087<br><br>» show all<br>TaxonomyUniProt
**Reference**	PMID:18563465
**Authors**	Jayasimha P, Nes WD
**Title**	Photoaffinity labeling and mutational analysis of 24-C-sterol methyltransferase defines the AdoMet binding site.
**Journal**	Lipids 43:681-93 (2008)<br>DOI:10.1007/s11745-008-3198-x
**Sequence**	[sce:YML008C]
**Reference**	PMID:20946868
**Authors**	Ganapathy K, Kanagasabai R, Nguyen TT, Nes WD

**Title**	Purification, characterization and inhibition of sterol C24-methyltransferase from Candida albicans.
**Journal**	Arch Biochem Biophys 505:194-201 (2011)<br>DOI:10.1016/j.abb.2010.10.008
**Sequence**	[cal:CAALFM_C302150CA]
**Reference**	PMID:10852933
**Authors**	Diener AC, Li H, Zhou W, Whoriskey WJ, Nes WD, Fink GR
**Title**	Sterol methyltransferase 1 controls the level of cholesterol in plants.
**Journal**	Plant Cell 12:853-70 (2000)<br>DOI:10.1105/tpc.12.6.853
**Sequence**	[ath:AT5G13710]

KEGG **ORTHOLOGY: K06287**

Help

**Entry**	K06287 KO
**Symbol**	yhdE
**Name**	nucleoside triphosphate pyrophosphatase [EC:3.6.1.-]
**Pathway**	map00240 Pyrimidine metabolism<br>map01100 Metabolic pathways<br>map01232 Nucleotide metabolism
**Reaction**	R00515 CTP diphosphohydrolase (diphosphate-forming)<br>R00662 uridine triphosphate pyrophosphohydrolase<br>R11323 dTTP diphosphohydrolase
**Brite**	KEGG Orthology (KO) [BR:ko00001]<br>09100 Metabolism<br>09104 Nucleotide metabolism

	00240 Pyrimidine metabolism<br>K06287 yhdE; nucleoside triphosphate pyrophosphatase
**Other DBs**	COG: COG0424
**Genes**	SCE: YOR111W<br><br>SEUB: DI49_4947<br><br>SPAO: SPAR_O02440<br><br>AGO: AGOS_AFL225W<br><br>ERC: Ecym_5418<br><br>KLA: KLLA0_F23628g<br><br>KMX: KLMA_60231<br><br>LTH: KLTH0D09240g<br><br>VPO: Kpol_505p13<br><br>ZRO: ZYRO0B09768g<br><br>» show all<br>TaxonomyUniProt
**Reference**	PMID:24210219
**Authors**	Tchigvintsev A, Tchigvintsev D, Flick R, Popovic A, Dong A, Xu X, Brown G, Lu W, Wu H, Cui H, Dombrowski L, Joo JC, Beloglazova N, Min J, Savchenko A, Caudy AA, Rabinowitz JD, Murzin AG, Yakunin AF
**Title**	Biochemical and structural studies of conserved Maf proteins revealed nucleotide pyrophosphatases with a preference for modified nucleotides.
**Journal**	Chem Biol 20:1386-98 (2013)<br>DOI:10.1016/j.chembiol.2013.09.011
**Sequence**	[sce:YOR111W] [eco:b3248] [bsu:BSU28050]

KEGG **ORTHOLOGY: K03380**

Help

**Entry**	K03380 KO
**Symbol**	E1.14.13.7
**Name**	phenol 2-monooxygenase (NADPH) [EC:1.14.13.7]
**Pathway**	map00623 Toluene degradation<br>map00627 Aminobenzoate degradation<br>map01120 Microbial metabolism in diverse environments
**Reaction**	R00815 phenol,NADPH:oxogen oxidoreductase (2-hydroxylating)<br>R03566 3-cresol,NADPH:oxogen oxidoreductase (2-hydroxylating)
**Brite**	KEGG Orthology (KO) [BR:ko00001]<br>09100 Metabolism<br>09111 Xenobiotics biodegradation and metabolism<br>00627 Aminobenzoate degradation<br>K03380 E1.14.13.7; phenol 2-monooxygenase (NADPH)<br>00623 Toluene degradation<br>K03380 E1.14.13.7; phenol 2-monooxygenase (NADPH)<br>Enzymes [BR:ko01000]<br>1. Oxidoreductases<br>1.14 Acting on paired donors, with incorporation or reduction of molecular oxygen<br>1.14.13 With NADH or NADPH as one donor, and incorporation of one atom of oxygen into the other donor<br>1.14.13.7 phenol 2-monooxygenase (NADPH)<br>K03380 E1.14.13.7; phenol 2-monooxygenase (NADPH)
**Other DBs**	COG: COG0654<br>GO: 0018662
**Genes**	PXB: 103942874<br>DHA: DEHA2C14784g<br>PIC: PICST_57166(PHH2)

	SPAA: SPAPADRAFT_59206<br><br>LEL: PVL30_004896<br><br>LBG: 92210592(LODBEIA_P53960)<br><br>COT: CORT_0D06110<br><br>CAL: CAALFM_CR07820WA(CaO19.3711) CAALFM_CR07940WA(CaO19.604)<br><br>CTP: CTRG_00423 CTRG_03102<br><br>CDU: CD36_33480 CD36_33600<br><br>» show all<br>TaxonomyUniProt
**Reference**	PMID:1429434
**Authors**	Kalin M, Neujahr HY, Weissmahr RN, Sejlitz T, Johl R, Fiechter A, Reiser J
**Title**	Phenol hydroxylase from Trichosporon cutaneum: gene cloning, sequence analysis, and functional expression in Escherichia coli.
**Journal**	J Bacteriol 174:7112-20 (1992)<br>DOI:10.1128/JB.174.22.7112-7120.1992
**Sequence**	[ag:AAA34202]

ORTHOLOGY: K00587

Help

**Entry**	K00587 KO
**Symbol**	ICMT, STE14
**Name**	protein-S-isoprenylcysteine O-methyltransferase [EC:2.1.1.100]
**Pathway**	map00900 Terpenoid backbone biosynthesis<br><br>map01110 Biosynthesis of secondary metabolites
**Reaction**	R04496 S-adenosyl-L-methionine:protein-C-terminal-S-farnesyl-L-cysteine O-methyltransferase

**Brite**	KEGG Orthology (KO) [BR:ko00001]<br>09100 Metabolism<br>09109 Metabolism of terpenoids and polyketides<br>00900 Terpenoid backbone biosynthesis<br>K00587 ICMT, STE14; protein-S-isoprenylcysteine O-methyltransferase<br>Enzymes [BR:ko01000]<br>2. Transferases<br>2.1 Transferring one-carbon groups<br>2.1.1 Methyltransferases<br>2.1.1.100 protein-S-isoprenylcysteine O-methyltransferase<br>K00587 ICMT, STE14; protein-S-isoprenylcysteine O-methyltransferase
**Other DBs**	COG: COG2020<br><br>GO: 0004671
**Genes**	HSA: 23463(ICMT)<br><br>PTR: 469782(ICMT)<br><br>PPS: 100978940(ICMT)<br><br>GGO: 101151352(ICMT)<br><br>PON: 100456656(ICMT)<br><br>PPYG: 129009264(ICMT)<br><br>NLE: 100583818(ICMT)<br><br>HMH: 116812840(ICMT)<br><br>SSYN: 129472492(ICMT)<br><br>MCC: 702925(ICMT)<br><br>» show all<br>TaxonomyUniProt
**Reference**	PMID:8289819
**Authors**	Sapperstein S, Berkower C, Michaelis S
**Title**	Nucleotide sequence of the yeast STE14 gene, which encodes farnesylcysteine carboxyl methyltransferase, and demonstration of its essential role in a-factor export.

**Journal**	Mol Cell Biol 14:1438-49 (1994)<br>DOI:10.1128/MCB.14.2.1438
**Sequence**	[sce:YDR410C]
**Reference**	PMID:17699599
**Authors**	Chen Y, McQuade KJ, Guan XJ, Thomason PA, Wert MS, Stock JB, Cox EC
**Title**	Isoprenylcysteine carboxy methylation is essential for development in Dictyostelium discoideum.
**Journal**	Mol Biol Cell 18:4106-18 (2007)<br>DOI:10.1091/mbc.E06-11-1006
**Sequence**	[ddi:DDB_G0272799]
**Reference**	PMID:18957507
**Authors**	Huizinga DH, Omosegbon O, Omery B, Crowell DN
**Title**	Isoprenylcysteine methylation and demethylation regulate abscisic acid signaling in Arabidopsis.
**Journal**	Plant Cell 20:2714-28 (2008)<br>DOI:10.1105/tpc.107.053389
**Sequence**	[ath:AT5G23320 AT5G08335]

KEGG **ORTHOLOGY: K20896**

Help

**Entry**	K20896 KO
**Symbol**	TENA_E
**Name**	formylaminopyrimidine deformylase / aminopyrimidine aminohydrolase [EC:3.5.1.- 3.5.99.-]
**Pathway**	map00730 Thiamine metabolism<br>map01100 Metabolic pathways

**Reaction**	R09993 4-amino-5-aminomethyl-2-methylpyrimidine aminohydrolase<br><br>R11313 N-formyl-4-amino-5-aminomethyl-2-methylpyrimidine amidohydrolase
**Brite**	KEGG Orthology (KO) [BR:ko00001]<br>09100 Metabolism<br>09108 Metabolism of cofactors and vitamins<br>00730 Thiamine metabolism<br>K20896 TENA_E; formylaminopyrimidine deformylase / aminopyrimidine aminohydrolase
**Other DBs**	COG: COG0819<br><br>GO: 0102281
**Genes**	OSA: 4332604<br><br>DOSA: Os03g0306900<br><br>OBR: 102722658<br><br>OGL: 127767501<br><br>BDI: 100834537<br><br>ATS: 109750418<br><br>TDC: 119285517<br><br>TAES: 123085664 123092365 123099968<br><br>HVG: 123450583<br><br>LPER: 127295625<br><br>» show all<br>TaxonomyUniProt
**Reference**	PMID:25014715
**Authors**	Zallot R, Yazdani M, Goyer A, Ziemak MJ, Guan JC, McCarty DR, de Crecy-Lagard V, Gerdes S, Garrett TJ, Benach J, Hunt JF, Shintani DK, Hanson AD
**Title**	Salvage of the thiamin pyrimidine moiety by plant TenA proteins lacking an active-site cysteine.

**Journal**	Biochem J 463:145-55 (2014)<br>DOI:10.1042/BJ20140522
**Sequence**	[ath:AT3G16990] [zma:100192786]

ORTHOLOGY: K00275

Help

**Entry**	K00275 KO
**Symbol**	pdxH, PNPO
**Name**	pyridoxamine 5'-phosphate oxidase [EC:1.4.3.5]
**Pathway**	map00750 Vitamin B6 metabolism<br>map01100 Metabolic pathways<br>map01120 Microbial metabolism in diverse environments<br>map01240 Biosynthesis of cofactors
**Module**	M00124 Pyridoxal phosphate biosynthesis, erythrose-4P => pyridoxal-P
**Reaction**	R00277 pyridoxamine-5'-phosphate:oxygen oxidoreductase (deaminating)<br>R00278 pyridoxine 5'-phosphate:oxygen oxidoreductase<br>R01710 pyridoxamine:oxygen oxidoreductase (deaminating)<br>R01711 pyridoxine:oxygen oxidoreductase (deaminating)
**Disease**	H01124 Pyridoxamine-5'-phosphate oxidase (PNPO) deficiency
**Brite**	KEGG Orthology (KO) [BR:ko00001]<br>09100 Metabolism<br>09108 Metabolism of cofactors and vitamins<br>00750 Vitamin B6 metabolism<br>K00275 pdxH, PNPO; pyridoxamine 5'-phosphate oxidase<br>Enzymes [BR:ko01000]<br>1. Oxidoreductases<br>1.4 Acting on the CH-NH2 group of donors<br>1.4.3 With oxygen as acceptor

	1.4.3.5 pyridoxal 5'-phosphate synthase<br>K00275 pdxH, PNPO; pyridoxamine 5'-phosphate oxidase
**Other DBs**	COG: COG0259<br><br>GO: 0004733
**Genes**	HSA: 55163(PNPO)<br><br>PTR: 749248(PNPO)<br><br>PPS: 100987061(PNPO)<br><br>GGO: 101123972(PNPO)<br><br>PON: 100443892(PNPO)<br><br>PPYG: 129016618(PNPO)<br><br>NLE: 100602483(PNPO)<br><br>HMH: 116474704(PNPO)<br><br>SSYN: 129470029(PNPO)<br><br>MCC: 694949(PNPO)<br><br>» show all<br>TaxonomyUniProt
**Reference**	PMID:12824491
**Authors**	Musayev FN, Di Salvo ML, Ko TP, Schirch V, Safo MK
**Title**	Structure and properties of recombinant human pyridoxine 5'-phosphate oxidase.
**Journal**	Protein Sci 12:1455-63 (2003)<br>DOI:10.1110/ps.0356203
**Sequence**	[hsa:55163]
**Reference**	PMID:15858270
**Authors**	Safo MK, Musayev FN, Schirch V
**Title**	Structure of Escherichia coli pyridoxine 5'-phosphate oxidase in a tetragonal crystal form: insights into the mechanistic pathway of the enzyme.

**Journal**	Acta Crystallogr D Biol Crystallogr 61:599-604 (2005)<br>DOI:10.1107/S0907444905005512
**Sequence**	[eco:b1638]

ORTHOLOGY: K01673

Help

**Entry**	K01673 KO
**Symbol**	cynT, can
**Name**	carbonic anhydrase [EC:4.2.1.1]
**Pathway**	map00910 Nitrogen metabolism<br>map01100 Metabolic pathways
**Reaction**	R00132 carbonate hydro-lyase (carbon-dioxide-forming)<br>R10092 carbonate hydro-lyase (carbon-dioxide-forming)
**Brite**	KEGG Orthology (KO) [BR:ko00001]<br>09100 Metabolism<br>09102 Energy metabolism<br>00910 Nitrogen metabolism<br>K01673 cynT, can; carbonic anhydrase<br>Enzymes [BR:ko01000]<br>4. Lyases<br>4.2 Carbon-oxygen lyases<br>4.2.1 Hydro-lyases<br>4.2.1.1 carbonic anhydrase<br>K01673 cynT, can; carbonic anhydrase
**Other DBs**	COG: COG0288<br>GO: 0004089
**Genes**	BFO: 118418545<br>BBEL: 109473612<br>SPU: 581001

	LVG: 121429566<br><br>AJJ: 139974687<br><br>APLC: 110981718<br><br>PMIN: 119724286<br><br>ARUN: 117299013<br><br>AAMU: 139944905<br><br>AFIL: 140160492<br><br>» show all<br>TaxonomyUniProt
**Reference**	PMID:15753099
**Authors**	Suarez Covarrubias A, Larsson AM, Hogbom M, Lindberg J, Bergfors T, Bjorkelid C, Mowbray SL, Unge T, Jones TA
**Title**	Structure and function of carbonic anhydrases from Mycobacterium tuberculosis.
**Journal**	J Biol Chem 280:18782-9 (2005)<br>DOI:10.1074/jbc.M414348200
**Sequence**	[mtu:Rv3588c]
**Reference**	PMID:1740425
**Authors**	Guilloton MB, Korte JJ, Lamblin AF, Fuchs JA, Anderson PM
**Title**	Carbonic anhydrase in Escherichia coli. A product of the cyn operon.
**Journal**	J Biol Chem 267:3731-4 (1992)<br>DOI:10.1016/S0021-9258(19)50586-5
**Sequence**	[eco:b0339]
**Reference**	PMID:20659325
**Authors**	Syrjanen L, Tolvanen M, Hilvo M, Olatubosun A, Innocenti A, Scozzafava A, Leppiniemi J, Niederhauser B, Hytonen VP, Gorr TA, Parkkila S, Supuran CT

**Title**	Characterization of the first beta-class carbonic anhydrase from an arthropod (Drosophila melanogaster) and phylogenetic analysis of beta-class carbonic anhydrases in invertebrates.
**Journal**	BMC Biochem 11:28 (2010)<br>DOI:10.1186/1471-2091-11-28
**Sequence**	[dme:Dmel_CG11967]

KEGG **ORTHOLOGY: K01522**

Help

**Entry**	K01522 KO
**Symbol**	FHIT
**Name**	bis(5'-adenosyl)-triphosphatase [EC:3.6.1.29]
**Pathway**	map00230 Purine metabolism<br>map01100 Metabolic pathways<br>map05222 Small cell lung cancer<br>map05223 Non-small cell lung cancer
**Reaction**	R00187 P1,P3-bis(5'-adenosyl)-triphosphate adenylohydrolase
**Disease**	H00013 Small cell lung cancer<br>H00014 Non-small cell lung cancer
**Brite**	KEGG Orthology (KO) [BR:ko00001]<br>09100 Metabolism<br>09104 Nucleotide metabolism<br>00230 Purine metabolism<br>K01522 FHIT; bis(5'-adenosyl)-triphosphatase<br>09160 Human Diseases<br>09162 Cancer: specific types<br>05222 Small cell lung cancer<br>K01522 FHIT; bis(5'-adenosyl)-triphosphatase<br>05223 Non-small cell lung cancer<br>K01522 FHIT; bis(5'-adenosyl)-triphosphatase

	Enzymes [BR:ko01000]<br>3. Hydrolases<br>3.6 Acting on acid anhydrides<br>3.6.1 In phosphorus-containing anhydrides<br>3.6.1.29 bis(5'-adenosyl)-triphosphatase<br>K01522 FHIT; bis(5'-adenosyl)-triphosphatase
**Other DBs**	GO: 0047710
**Genes**	HSA: 2272(FHIT)<br>PTR: 747260(FHIT)<br>PPS: 100985630(FHIT)<br>GGO: 101139888(FHIT)<br>PPYG: 129032485(FHIT)<br>NLE: 100587886(FHIT)<br>HMH: 116466611(FHIT)<br>SSYN: 129471356(FHIT)<br>MCC: 700974(FHIT)<br>MCF: 102118172(FHIT)<br>» show all<br>TaxonomyUniProt
**Reference**	PMID:8794732
**Authors**	Barnes LD, Garrison PN, Siprashvili Z, Guranowski A, Robinson AK, Ingram SW, Croce CM, Ohta M, Huebner K
**Title**	Fhit, a putative tumor suppressor in humans, is a dinucleoside 5',5"'-P1,P3-triphosphate hydrolase.
**Journal**	Biochemistry 35:11529-35 (1996)<br>DOI:10.1021/bi961415t
**Sequence**	[hsa:2272]
**Reference**	PMID:9576908

**Authors**	Pace HC, Garrison PN, Robinson AK, Barnes LD, Draganescu A, Rosler A, Blackburn GM, Siprashvili Z, Croce CM, Huebner K, Brenner C
**Title**	Genetic, biochemical, and crystallographic characterization of Fhit-substrate complexes as the active signaling form of Fhit.
**Journal**	Proc Natl Acad Sci U S A 95:5484-9 (1998)<br>DOI:10.1073/pnas.95.10.5484
**Sequence**	[hsa:2272]

ORTHOLOGY: K01505

Help

**Entry**	K01505 KO
**Symbol**	E3.5.99.7
**Name**	1-aminocyclopropane-1-carboxylate deaminase [EC:3.5.99.7]
**Pathway**	map00270 Cysteine and methionine metabolism<br><br>map01100 Metabolic pathways
**Reaction**	R00997 1-aminocyclopropane-1-carboxylate aminohydrolase (isomerizing)
**Brite**	KEGG Orthology (KO) [BR:ko00001]<br>09100 Metabolism<br>09105 Amino acid metabolism<br>00270 Cysteine and methionine metabolism<br>K01505 E3.5.99.7; 1-aminocyclopropane-1-carboxylate deaminase<br>Enzymes [BR:ko01000]<br>3. Hydrolases<br>3.5 Acting on carbon-nitrogen bonds, other than peptide bonds<br>3.5.99 In other compounds<br>3.5.99.7 1-aminocyclopropane-1-carboxylate deaminase<br>K01505 E3.5.99.7; 1-aminocyclopropane-1-carboxylate deaminase
**Other DBs**	COG: COG2515<br><br>GO: 0008660
**Genes**	ZMK: HG535_0C00220

	CLU: CLUG_04006<br>CLUS: A9F13_01g01166<br>PKZ: C5L36_0E04640<br>NCR: NCU04657<br>NTE: NEUTE1DRAFT77478(NEUTE1DRAFT_77478)<br>SMP: 10804801(SMAC4_08345)<br>PAN: PODANSg8093<br>PBEL: QC761_203080<br>PPSD: 87907103<br>» show all<br>TaxonomyUniProt
**Reference**	PMID:15588698
**Authors**	Hontzeas N, Zoidakis J, Glick BR, Abu-Omar MM
**Title**	Expression and characterization of 1-aminocyclopropane-1-carboxylate deaminase from the rhizobacterium Pseudomonas putida UW4: a key enzyme in bacterial plant growth promotion.
**Journal**	Biochim Biophys Acta 1703:11-9 (2004)<br>DOI:10.1016/j.bbapap.2004.09.015
**Sequence**	[ppuu:PputUW4_04154]

ORTHOLOGY: K01555

Help

**Entry**	K01555	KO
**Symbol**	FAH, fahA	
**Name**	fumarylacetoacetase [EC:3.7.1.2]	
**Pathway**	map00350 Tyrosine metabolism	

	map00643 Styrene degradation<br><br>map01100 Metabolic pathways<br><br>map01120 Microbial metabolism in diverse environments
**Module**	M00044 Tyrosine degradation, tyrosine => homogentisate
**Reaction**	R01364 4-fumarylacetoacetate fumarylhydrolase
**Disease**	H00165 Tyrosinemia
**Brite**	KEGG Orthology (KO) [BR:ko00001]<br>09100 Metabolism<br>09105 Amino acid metabolism<br>00350 Tyrosine metabolism<br>K01555 FAH, fahA; fumarylacetoacetase<br>09111 Xenobiotics biodegradation and metabolism<br>00643 Styrene degradation<br>K01555 FAH, fahA; fumarylacetoacetase<br>09180 Brite Hierarchies<br>09183 Protein families: signaling and cellular processes<br>04147 Exosome<br>K01555 FAH, fahA; fumarylacetoacetase<br>Enzymes [BR:ko01000]<br>3. Hydrolases<br>3.7 Acting on carbon-carbon bonds<br>3.7.1 In ketonic substances<br>3.7.1.2 fumarylacetoacetase<br>K01555 FAH, fahA; fumarylacetoacetase<br>Exosome [BR:ko04147]<br>Exosomal proteins<br>Exosomal proteins of other body fluids (saliva and urine)<br>K01555 FAH, fahA; fumarylacetoacetase
**Other DBs**	GO: 0004334
**Genes**	HSA: 2184(FAH)<br><br>PTR: 467738(FAH)<br><br>PPS: 100969245(FAH)

	GGO: 101126860(FAH)<br>PON: 100437222(FAH)<br>PPYG: 129014704(FAH)<br>NLE: 100589446(FAH)<br>HMH: 116482605(FAH)<br>SSYN: 129482164(FAH)<br>MCC: 712716(FAH)<br>» show all<br>TaxonomyUniProt
**Reference**	PMID:9417084
**Authors**	Fernandez-Canon JM, Penalva MA
**Title**	Characterization of a fungal maleylacetoacetate isomerase gene and identification of its human homologue.
**Journal**	J Biol Chem 273:329-37 (1998)<br>DOI:10.1074/jbc.273.1.329
**Sequence**	[ani:ANIA_01896]

ORTHOLOGY: K01233

Help

**Entry**	K01233 KO
**Symbol**	csn
**Name**	chitosanase [EC:3.2.1.132]
**Pathway**	map00520 Amino sugar and nucleotide sugar metabolism<br>map01100 Metabolic pathways
**Reaction**	R02833 chitosan N-acetylglucosaminohydrolase

**Brite**	KEGG Orthology (KO) [BR:ko00001]<br>09100 Metabolism<br>09107 Glycan biosynthesis and metabolism<br>00520 Amino sugar and nucleotide sugar metabolism<br>K01233 csn; chitosanase<br>Enzymes [BR:ko01000]<br>3. Hydrolases<br>3.2 Glycosylases<br>3.2.1 Glycosidases, i.e. enzymes that hydrolyse O- and S-glycosyl compounds<br>3.2.1.132 chitosanase<br>K01233 csn; chitosanase
**Other DBs**	GO: 0016977
**Genes**	BGT: 106060432 106073034<br><br>FVN: FVRRES_04507<br><br>FVR: FVEG_01778<br><br>FOX: FOXG_02920<br><br>FMU: J7337_008139<br><br>TRE: TRIREDRAFT_70341<br><br>TRR: M419DRAFT_13025<br><br>TATV: 25775788(TrAtP1_005186) 25785205(TrAtP1_009221) 25785498(TrAtP1_013230)<br><br>TASP: 36610278(TrAFT101_004655) 36612199(TrAFT101_009547) 36615514(TrAFT101_004570)<br><br>MAW: 19252408(J3458_015444)<br><br>» show all<br>TaxonomyUniProt
**Reference**	PMID:11065371
**Authors**	Rivas LA, Parro V, Moreno-Paz M, Mellado RP

**Title**	The Bacillus subtilis 168 csn gene encodes a chitosanase with similar properties to a streptomyces enzyme.
**Journal**	Microbiology 146 (Pt 11):2929-36 (2000)<br>DOI:10.1099/00221287-146-11-2929
**Sequence**	[bsu:BSU26890]
